# Homologs of the Escherichia coli F Element Protein TraR, Including Phage Lambda Orf73, Directly Reprogram Host Transcription

**DOI:** 10.1128/mbio.00952-22

**Published:** 2022-05-18

**Authors:** Saumya Gopalkrishnan, Wilma Ross, Madeline S. Akbari, Xintian Li, James R. J. Haycocks, David C. Grainger, Donald L. Court, Richard L. Gourse

**Affiliations:** a University of Wisconsin—Madison, Department of Bacteriology, Madison, Wisconsin, USA; b University of Birmingham, Institute of Microbiology and Infection, School of Biosciences, Edgbaston, Birmingham, United Kingdom; c RNA Biology Laboratory, Center for Cancer Research, The National Cancer Institute at Frederick, Frederick, Maryland, USA; Massachusetts Institute of Technology

**Keywords:** DksA homologs, RNA polymerase, TraR homologs, bacteriophage lambda Orf73, regulation of transcription

## Abstract

Bacterial cells and their associated plasmids and bacteriophages encode numerous small proteins of unknown function. One example, the 73-amino-acid protein TraR, is encoded by the transfer operon of the conjugative F plasmid of Escherichia coli. TraR is a distant homolog of DksA, a protein found in almost all proteobacterial species that is required for ppGpp to regulate transcription during the stringent response. TraR and DksA increase or decrease transcription initiation depending on the kinetic features of the promoter by binding directly to RNA polymerase without binding to DNA. Unlike DksA, whose full activity requires ppGpp as a cofactor, TraR is fully active by itself and unaffected by ppGpp. TraR belongs to a family of divergent proteins encoded by proteobacterial bacteriophages and other mobile elements. Here, we experimentally addressed whether other members of the TraR family function like the F element-encoded TraR. Purified TraR and all 5 homologs that were examined bound to RNA polymerase, functioned at lower concentrations than DksA, and complemented a *dksA*-null strain for growth on minimal medium. One of the homologs, λ Orf73, encoded by bacteriophage lambda, was examined in greater detail. λ Orf73 slowed host growth and increased phage burst size. Mutational analysis suggested that λ Orf73 and TraR have a similar mechanism for inhibiting rRNA and r-protein promoters. We suggest that TraR and its homologs regulate host transcription to divert cellular resources to phage propagation or conjugation without induction of ppGpp and a stringent response.

## INTRODUCTION

The majority of small proteins (defined here arbitrarily as less than 75 amino acids in length) have been identified simply as short open reading frames of unknown function. However, in recent years, a substantial number of open reading frames have been validated by genetic and biochemical approaches as having important functions in bacterial systems (1–4).

One of these small proteins, TraR, is a 73-amino-acid polypeptide encoded by the Escherichia coli conjugation plasmid F. TraR is a distant homolog of the transcription factor DksA, corresponding in structure to the C-terminal half of the 151-amino-acid DksA protein (5, 6). DksA is found in almost all proteobacteria and binds to RNA polymerase (RNAP) in conjunction with the signaling nucleotides ppGpp and pppGpp (abbreviated here as ppGpp) during the stringent response, the highly conserved nutritional stress response found in nearly all bacteria (7). ppGpp also binds to many other enzymes to regulate metabolic processes during the cell’s response to nutrient deprivation (8, 9).

DksA interacts directly with residues in the secondary channel of RNAP, and ppGpp binds to a pocket at the interface of DksA and the β′ subunit (10, 11). DksA and ppGpp together alter the conformation of RNAP, thereby regulating hundreds of promoters in E. coli that utilize the primary sigma factor, σ^70^, including promoters for rRNA, ribosomal proteins, and amino acid biosynthesis enzymes (12). They also regulate promoters utilizing the extracytoplasmic stress response sigma factor, σ^E^ (13) and promoters that regulate expression of the general stress response sigma factor, σ^S^ (14). ppGpp is synthesized in response to nutrient limitation, whereas conjugation of the F plasmid encoding TraR occurs optimally during exponential-phase growth in rich medium, a process not known to induce ppGpp (15). Therefore, one hypothesis is that TraR might have evolved in part to turn off transcription of genes needed for cell growth temporarily and to turn on genes needed to repair disruptions of the cell membrane during conjugation (6, 16).

Recent biochemical and structural analyses have informed us about the mechanisms by which TraR mimics the combined effects of DksA and ppGpp on RNAP (6, 11, 17, 18), activating or inhibiting transcription initiation depending on the kinetic properties of the promoter. TraR does not contain matches to the residues in DksA that bind ppGpp, and furthermore, the residues in β′ that interact with ppGpp when DksA is bound in the secondary channel of RNAP are sterically occluded by binding of TraR. As a result, ppGpp does not bind to the TraR-RNAP complex or contribute to the effects of TraR on transcription (6). Rather, cryo-electron microscopy (cryo-EM) approaches (17, 18) have shown that TraR by itself induces major conformational changes in RNAP that were not evident in earlier X-ray studies because of crystal packing constraints (11). These conformational changes include an ~18° rotation of the β lobe and β Si1 subdomains of RNAP that move together as a rigid body, as well as a large reorientation of the β′Si3 insertion. These changes widen the main channel of RNAP and weaken its interactions with σ region 1.1.

TraR inhibits transcription through multiple effects on promoter complexes. Namely, it stabilizes an early intermediate along the pathway to open complex formation, thereby lowering the rate of opening at promoters where this step is rate limiting. It also reduces the stability of later intermediates, further reducing open complex formation at promoters that make intrinsically unstable complexes. Finally, a TraR-induced β′ shelf rotation leads to formation of a kink in the bridge helix that sterically prevents proper positioning of the template strand DNA in the open complex. Because of the instability of open complexes formed by RNAP-TraR with inhibited promoters, most RNAP and bound TraR dissociate from the DNA before TraR dissociates from the RNAP. The continued presence of TraR in any remaining promoter-bound complexes prevents initiation by preventing template strand placement in the active site. (6, 17–19). Inhibition is largely independent of the effects of TraR on σ^70^_1.1_, since inhibited promoters (e.g., rRNA promoters) largely outcompete σ^70^_1.1_ for the main channel even without the help of TraR.

At a different set of promoters, interactions between TraR and RNAP activate transcription. The conformational changes that widen the main channel of RNAP and weaken its interactions with σ region 1.1 (see above) help promoter DNA compete with σ region 1.1 for the main channel. TraR thereby increases transcription from promoters that are rate-limited at this step in open complex formation (6, 17, 18). Once formed, open complexes at activated promoters are stable enough that TraR dissociates before the DNA-RNAP complex decays. Thus, this promoter class is not inhibited by the continued presence of TraR (17–19).

In this study, we extended our studies of TraR, and we investigated five homologs found in bacteriophages that infect gamma proteobacteria. Previously, we computationally predicted an extensive list of homologs with lengths similar to that of TraR (6) and used the ConSurf webserver (20) to identify highly conserved residues. Most of the conserved residues were in three distinct regions of the protein. Biochemical analyses demonstrated that these regions were important for transcriptional regulation by TraR (6).

We found that the five homologs have properties similar although not identical to those of TraR. We showed that the homologs complement an E. coli *dksA*-null strain, and the purified proteins bind to E. coli RNAP and regulate transcription initiation *in vitro*. We then focused on one of the homologs, Orf73 from bacteriophage λ. Previous studies have variously implicated this protein in inhibition of the initiation of host replication (21) or in the phage lysis-lysogeny decision (22), but the mechanism(s) responsible has not been identified. We show here that λ Orf73 has strong effects on host cell transcription *in vitro* and *in vivo* and increases phage production. Our results highlight a role for λ Orf73 in the lytic cycle of phage lambda and support a model in which TraR and its homologs inhibit host transcription under conditions that do not generate a stringent response. This shifts the focus of the cell’s transcription program from synthesis of the host translation machinery to transcription of phage or plasmid genes.

## RESULTS

### TraR homologs complement a Δ*dksA* strain for growth on minimal medium lacking amino acids.

**FIG 1:**
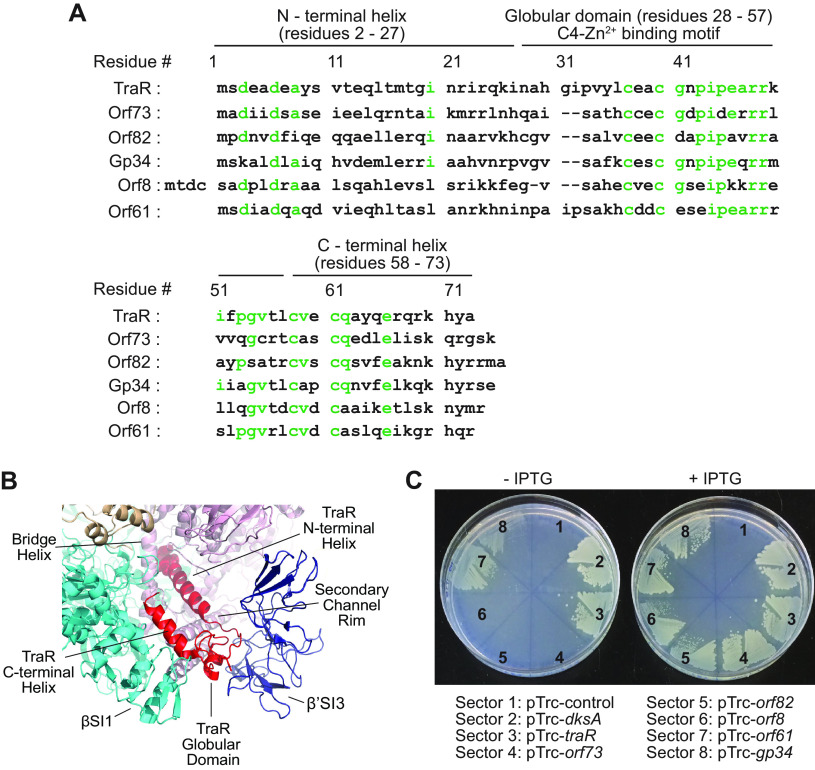
F element-encoded TraR and five TraR homologs. (A) Amino acid sequence alignment of E. coli TraR and five of its homologs: Orf73 from phage lambda, Orf82 from *Enterobacteria* phage P2, Gp34 from Xenorhabdus bovienii, Orf8 from *Vibrio* phage VHML, and Orf61 from *Vibrio* phage VP882. Three TraR structural elements, identified from the cryo-EM structure of the TraR-RNAP complex (17), are indicated above the TraR residue numbers. Amino acid residues shown in green indicate highly conserved residues among 200 putative TraR homologs analyzed using the ConSurf web server (6). (B) Model of TraR-RNAP complex showing regions of TraR interactions with sections of RNAP (adapted from PDB 6N58) (17). TraR, red, with residues D3 and D6 shown as spheres; RNAP β subunit, cyan; β′ subunit, pink; β′Si3 insertion, dark blue; σ^70^ subunit, light brown. (C) Growth of an E. coli Δ*dksA* strain containing plasmids for expression of DksA, TraR, or its homologs from the pTrc promoter on minimal medium lacking all amino acids, either with (left) or without (right) IPTG. Sectors contain strains with (1) empty vector (RLG14718), (2) DksA (RLG15137), (3) TraR (RLG14719), (4) λ Orf73 (RLG15139), (5) P2 Orf82 (RLG15140), (6) VHML Orf8 (RLG15169), (7) VP882 Orf61 (RLG15162), and (8) *X. bovienii* Gp34 (RLG15141).

Although we reported previously that TraR-like proteins are found throughout the bacterial domain, further analysis indicated that homologs with a length similar to that of TraR are found primarily in the *Proteobacteria* and their associated bacteriophages (Fig. S1). Bioinformatic analysis of 200 TraR-like proteins identified three regions of highest conservation (residues in green in the TraR sequence in Fig. 1A), and substitutions introduced into these regions resulted in defects in TraR function (6).

10.1128/mbio.00952-22.1 FIG S1 BLAST searches identified DksA/TraR homologs in the C4-type zinc finger family. Homologs in this family vary in size, being similar in length to TraR or DksA or longer or shorter. We limited our analysis to ~200 representative proteins similar in length (69 to 75 residues) to TraR (which contains 73 amino acids). The group of 200 TraR-length homologs were encoded primarily in genomic sequences from the classes *Alphaproteobacteria*, *Betaproteobacteria*, *Gammaproteobacteria*, and Deltaproteobacteria of the phylum *Proteobacteria* and in some bacteriophage genomes (6). Three representative bacterial species for each class that contain homologs are shown, and several bacteriophages from gamma proteobacteria are indicated for illustration. The genomic context in which the homologs appear was not identified by the BLAST searches, but we suggest that the homologs are likely encoded by integrated prophage or by plasmids. Specific phages from *Alpha*-, *Beta*-, or Deltaproteobacteria that contain TraR homologs have not been characterized to our knowledge. The tree was constructed using phyloT (http://phylot.biobyte.de/). Download FIG S1, EPS file, 1.3 MB. Copyright © 2022 Gopalkrishnan et al. 2022 Gopalkrishnan et al. https://creativecommons.org/licenses/by/4.0/ This content is distributed under the terms of the Creative Commons Attribution 4.0 International license.

The cryo-EM structure of the TraR-RNAP complex (17) identified three structural elements in TraR that interact with RNAP, corresponding to the three regions of highest conservation described above (Fig. 1B). The N-terminal helix contains a conserved DxxDxA motif critical for function (6) that interacts with the active-site region of RNAP (17). The central structural element is a globular domain that includes a C4 zinc binding motif and residues that interact with RNAP at the entrance to the secondary channel and with the β′Si3 sequence insertion. The C-terminal helix of TraR contains residues that interact with the β Si1 sequence insertion in the RNAP β lobe (17).

Based on this information, we analyzed five predicted TraR homologs from phages that infect gamma proteobacteria, including Orf73 from bacteriophage λ, Orf82 from *Enterobacterium* bacteriophage P2, Orf8 from Vibrio harveyi phage VHML, Orf61 from Vibrio parahaemolyticus phage VP882, and Gp34 from the genome of Xenorhabdus bovienii. λ and P2 are well-studied E. coli phages (23, 24). VHML and VP882 are previously studied *Vibrio* phages (25, 26). Gp34 is encoded by a likely prophage in the genome of *X. bovienii*, a symbiotic bacterium (27). Very little is known about the functions of the five homologs.

The amino acid sequences of the five homologs and TraR are aligned in Fig. 1A. These homologs contain many matches (shown in green) to the most highly conserved residues in our previously published ConSurf analysis of the 200 homologs (6), but they differ in other regions and are 32% to 49% identical and 45% to 63% similar to TraR overall. Nevertheless, the predicted 3-dimensional structures of the homologs constructed using the RaptorX modeling program (28) are similar to the structure of TraR (Fig. S2).

10.1128/mbio.00952-22.2 FIG S2 Structural models of TraR homologs. Models were generated using the RaptorX protein structure prediction program (http://raptorx.uchicago.edu/StructurePrediction/), using the web server-defined best structural templates (either PDB 5W1SM [TraR] or 1TJLA [DksA]). Models for four of the five TraR homologs resembled the structures of TraR (or the C-terminal half of DksA). The λ Orf73 model based on 1TJLA was largely disordered (*p*-value, 1.7e−01). Substitutions for either of two cysteine residues in λ Orf73 (C38 or C55 or both) (Fig. 1A) resulted in good structure predictions based on either 5W1SM or 1TJLA (residue numbers refer to the corresponding positions in TraR). (A) RaptorX-generated structure prediction for TraR (red) based on crystal structure 5W1SM (*p*-value, 2.67e−04). (B) λ Orf73 C38A variant (green) based on 5W1SM (*p*-value, 5.22e−04). (C) Orf82 (blue) based on 1TJLA (*p*-value, 2.81e−04). (D) Gp34 (purple) based on 5W1SM (*p*-value, 6.96 e−04). (E) Orf8 (cyan) based on 1TJLA (*p*-value, 3.58e−04). (F) Orf61 (yellow) based on 5W1SM (*p*-value, 2.74 e−04). Download FIG S2, EPS file, 2.7 MB. Copyright © 2022 Gopalkrishnan et al. 2022 Gopalkrishnan et al. https://creativecommons.org/licenses/by/4.0/ This content is distributed under the terms of the Creative Commons Attribution 4.0 International license.

TraR complements an E. coli strain lacking DksA (Δ*dksA*) for growth on minimal medium without amino acids (5, 6). To determine whether the similarity in sequences and predicted structures of the homologs was indicative of TraR-like functional properties, we first tested whether homolog genes fused to a *trc* promoter on a plasmid would complement a Δ*dksA* host. When IPTG (isopropyl-β-d-thiogalactopyranoside) was not added to induce the *trc* promoter on the plasmid (i.e., with only basal expression of the TraR homolog), only DksA and TraR fully complemented the Δ*dksA* host. Some limited growth was also observed with VP882 Orf61 and *X. bovienii* Gp34 (Fig. 1C). However, when expression from the plasmid was induced with IPTG, each of the five homologs (and TraR and DksA) fully complemented the Δ*dksA* strain. Therefore, we purified the proteins and tested whether each affected transcription *in vitro* to determine whether effects on transcription likely accounted for the complementation.

### Purified TraR and its homologs bind to RNAP and inhibit transcription from rRNA and ribosomal protein promoters.

**FIG 2:**
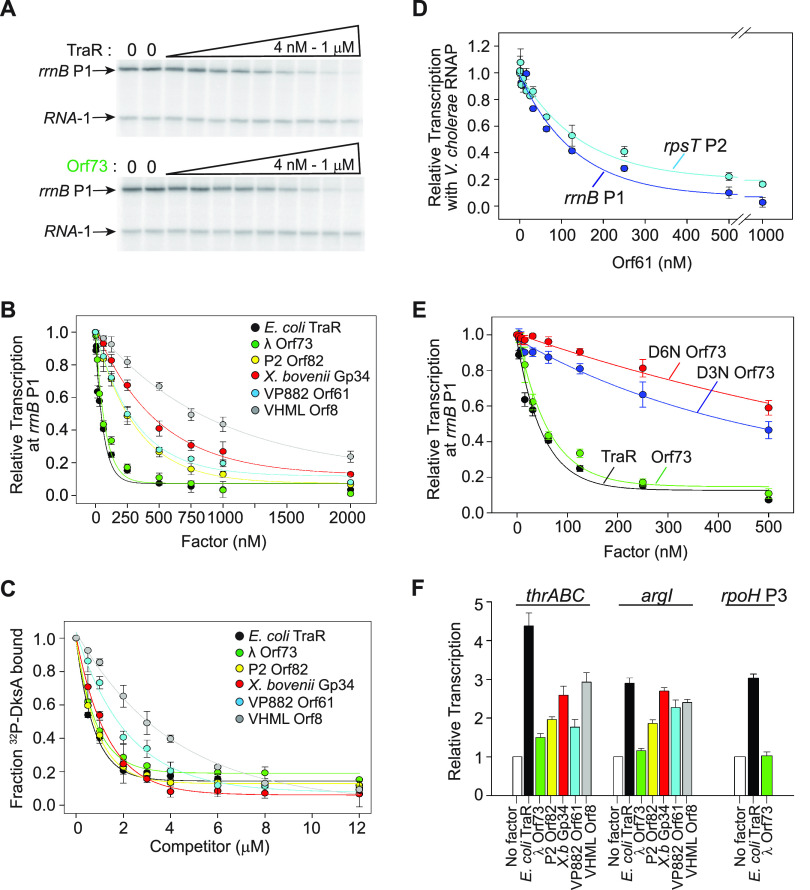
Regulation of transcription by TraR and TraR homologs *in vitro*. (A) Representative gel showing the transcripts from multiround transcription assays with the E. coli *rrnB* P1 promoter (pRLG13065) and the RNA-1 promoter by E. coli RNAP in the presence or absence of the indicated concentrations of TraR or λ Orf73. RNA-1 is a plasmid-encoded promoter involved in control of plasmid copy number. (B) Inhibition of *rrnB P1* transcription by a range of concentrations (4 nM to 2,000 nM) of TraR and each of the homologs from experiments like that reported in panel A. Transcription was normalized to that in the absence of factor (1.0 at 0 nM factor). IC_50_s for inhibition by each factor are provided in the text. Means and standard deviations from three independent experiments are shown. (C) Binding of each TraR homolog to E. coli RNAP. Binding was determined by a competition assay measuring the effect of each unlabeled homolog on binding of ^32^P-HMK-DksA to RNAP, as described in the text. The fraction of DksA bound at each concentration of factor was normalized to that in the absence of the factor. Means and standard deviations from three independent experiments are shown. (D) Inhibition of transcription from the V. cholerae *rrnB* P1 (pRLG15667) and *rpsT* P2 (pRLG15668) promoters with V. cholerae RNAP by VP882 Orf61 (4 nM to 1,000 nM). Transcription at each concentration of VP882 Orf61 was normalized to transcription in the absence of VP882 Orf61 (1.0 at 0 nM TraR homolog). Means and standard deviations from three independent experiments are shown. (E) Inhibition of E. coli *rrnB P1* (pRLG13065) transcription by Orf73 variants with substitution D3N or D6N in the DxxDxA motif. For comparison, inhibition is also shown for wild-type TraR and Orf73. Averages and standard deviations from three independent experiments are indicated. (F) Activation of transcription of the *thrABC* (pRLG5073), *argI* (pRLG13098), and *rpoH* P3 (pRLG13068) promoters by TraR or the indicated homologs. Transcription was performed as for panel B using a range of concentrations of factor (4 nM to 4,000 nM), but for clarity, bars indicate activation only at 250 nM, the concentration at which maximum activation was observed, relative to transcription in the absence of factor. Means and standard deviations from three independent experiments are shown.

To determine whether the 5 homologs functioned like TraR, the proteins were purified and tested in an *in vitro* transcription assay containing E. coli RNAP. It was shown previously that DksA/ppGpp and TraR regulate transcription from rRNA promoters that account for the majority of cellular transcription in moderately growing to fast-growing cells (6, 7). The 50% inhibitory concentrations (IC_50_s) of TraR and TraR/ppGpp were the same (6). Therefore, we first tested the effects of the TraR homologs on the rRNA promoter *rrnB* P1. The effects of increasing concentrations of TraR or a representative TraR homolog, λ Orf73, are shown in the transcription gel pictured in Fig. 2A. Each of the factors inhibited *rrnB* P1 transcription (Fig. 2B), but they did not inhibit transcription from the plasmid-encoded RNA-1 promoter (Fig. 2A). The IC_50_ for inhibition of *rrnB* P1 differed among the homologs. λ Orf73 had an IC_50_ very similar to that of TraR (~50 nM), whereas higher concentrations of the other homologs were required for inhibition (IC_50_s: P2 Orf82, 250 nM; VP882 Orf61, 270 nM; *X. bovienii* Gp34, 415 nM; VHML Orf8, 800 nM). As observed previously for TraR (6), the homologs inhibited transcription in the absence of ppGpp. The differences in IC_50_s among the homologs could reflect weaker affinities for E. coli RNAP and/or different effects on RNAP conformation, particularly since three of the homologs derived from non-E. coli hosts (VHML Orf8 and VP882 Orf61 from *Vibrio* phages and Gp34 from *X. bovienii*). We also found that TraR and Orf73 inhibited transcription from the promoter for ribosomal protein S20, *rpsT* P2 (Fig. S3).

10.1128/mbio.00952-22.3 FIG S3 Inhibition of transcription of *rpsT* P2 (pRLG14658) by λ Orf73 variants with substitutions in the DxxDxA motif, by wild-type λ Orf73, or by TraR. For comparison, inhibition of transcription of the *rrnB* P1 promoter by λ Orf73, TraR, and λ Orf73 variants is shown in Fig. 2E. The IC_50_ for inhibition of *rpsT* P2 by wild-type TraR (black circles) and Orf73 (green circles) was ~65 nM, by D3N Orf73 was ~650 nM (blue circles), and by D6N Orf73 (red circles) was ~1.2 μM. Assays were performed as for Fig. 2A and B. Means and standard deviations from three independent experiments are shown. Download FIG S3, EPS file, 1.1 MB. Copyright © 2022 Gopalkrishnan et al. 2022 Gopalkrishnan et al. https://creativecommons.org/licenses/by/4.0/ This content is distributed under the terms of the Creative Commons Attribution 4.0 International license.

To address whether the relative function of the different homologs correlated with their binding affinity for E. coli RNAP, we tested RNAP binding using an *in vitro* competition assay (Fig. 2C) (6). In this assay, increasing amounts of unlabeled TraR or TraR homolog were added to ^32^P-labeled E. coli DksA and E. coli RNAP, and the fraction of DksA bound to RNAP was compared to the fraction bound without the factor. The fraction of DksA bound was quantified from the amount of ^32^P-labeled DksA cleavage product produced by hydroxyl radicals generated by Fe^2+^ replacement of Mg^2+^ at the RNAP active site (29). The concentration dependence of competition by each factor (Fig. 2C) indicated that 3 of the homologs competed similarly to TraR for binding of DksA to RNAP (in the range of 0.65 to 1 μM protein for half maximal inhibition of DksA binding; λ Orf73, P2 Orf82, and *X. bovienii* Gp34), while the homologs from the two *Vibrio* phages competed less well (half-maximal inhibition of DksA binding of 1.7 μM for VP882 Orf61 and 3 μM for VHML Orf8). These experiments also showed that despite somewhat varying affinities, the homologs bound to the same site on RNAP as DksA and TraR.

For 3 of the homologs and TraR itself, the degree of inhibition of transcription from *rrnB* P1 (Fig. 2B) correlated with binding to RNAP (Fig. 2B and C). TraR and λ Orf73 had the lowest IC_50_s for inhibition, and Orf73 competed similarly to TraR for DksA binding to RNAP, while the two *Vibrio* phage proteins, VP882 Orf61 and VHML Orf8, had higher IC_50_s for inhibition and competed with DksA binding less well than TraR. These results suggested that weaker binding to E. coli RNAP than *Vibrio* RNAP might account, at least in part, for the reduced transcription inhibition by the *Vibrio* TraR homologs.

We found that inhibition by *Vibrio* Orf61 was 2- to 3-fold greater using purified *Vibrio* RNAP and the *Vibrio rrnB* P1 promoter (IC_50_ ~ 100 nM) than inhibition by Orf61 of the E. coli *rrnB* P1 promoter using E. coli RNAP by Orf61 (IC_50_ ~270 nM) (Fig. 2D), consistent with differences in affinity of Orf61 for the RNAPs and/or differences in the *rrnB* promoters from E. coli and *Vibrio.* We conclude that Orf61 functions effectively with its cognate RNAP to inhibit transcription from *rrnB* and also from the *Vibrio* S20 ribosomal protein promoter *rpsT* P2 (Fig. 2D), suggesting that effects of Orf61 on host transcription potentially could play a role in the growth of its phage VP882.

TraR contains two highly conserved aspartate residues, D3 and D6, in the DxxDxA motif near the N terminus of the protein (Fig. 1A) that are required for TraR function (6) (see Discussion). These residues are analogous to the two aspartates at the tip of the coiled coil of DksA that are essential for DksA function but not for DksA binding to RNAP (30). To address whether the homologs affected transcription by a mechanism related to that used by TraR (and DksA), we tested the effects of substitutions for these residues on transcription *in vitro* in one of the homologs, λ Orf73. Purified λ Orf73 strongly inhibited transcription from the E. coli *rrnB* P1 and *rpsT* P2 promoters, but the λ Orf73 variants containing either D3N or D6N inhibited transcription much less well than wild-type λ Orf73 (Fig. 2E and Fig. S3A). The requirement for the N-terminal aspartate residues in both λ Orf73 and TraR is consistent with the model that they utilize the same mechanism for inhibiting transcription. Given that both Gp34 and Orf82 bound as well as TraR to E. coli RNAP (Fig. 2B and C), their lower activities could be due in part to the less-than-perfect conservation of the DxxDxA motif in these two TraR homologs, i.e., a missing D3 in X. *bovienii* Gp34 and a missing A8 in Orf82 (Fig. 1A). Additionally, an N-terminal extension in VHML Orf8 could contribute to its lower activity.

### Activation of transcription by purified TraR and its homologs.

DksA and TraR inhibit transcription from ribosomal promoters like *rrnB* P1 and *rpsT* P2, but they also activate transcription of amino acid biosynthesis promoters like *thrABC* and *argI* and the heat shock promoter *rpoH* P3, promoters with kinetic properties very different from those for the ribosomal genes (6, 16). With the exception of λ Orf73, the homologs increased transcription from the *thrABC* and *argI* promoters from ~2- to 4-fold (Fig. 2F). Unlike with DksA (6), the observed activation with TraR did not require ppGpp. However, little or no activation of the *thrABC*, *argI*, and *rpoH* P3 promoters by λ Orf73 was observed, even though Orf73 strongly inhibited *rrnB* P1 (Fig. 2B) and *rpsT* P2 (Fig. S3). Given the limited numbers of promoters tested so far, it is premature to conclude that Orf73 is mechanistically incapable of activating transcription (see also Discussion).

### λ Orf73 increases λ phage yield.

**FIG 3:**
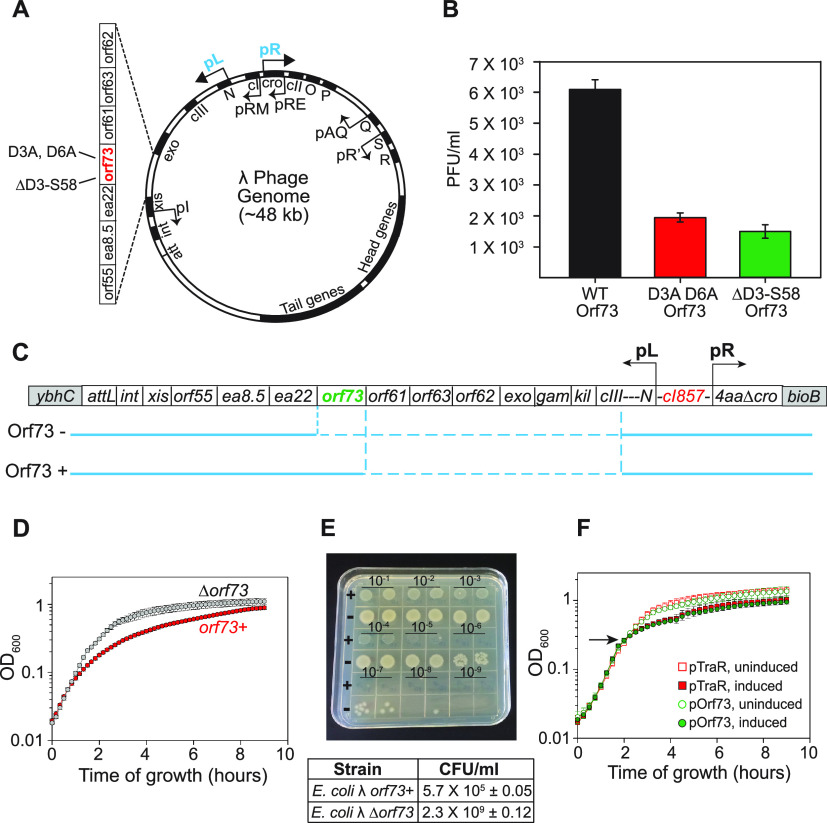
λ *orf73* increases phage production and reduces host growth rate. (A) Schematic map of the lambda genome, showing locations of the major promoters and an expanded map of the genes between *exo* and *xis*. Orf73 variants D3A, D6A, and ΔD3–S58, used for panel B, are also indicated. (B) Orf73 specifically increases the efficiency of λ phage particle formation. λ lysogens contained either wild-type *orf73* (RLG15500; *orf73*+, black bar), an *orf73* gene with D3A and D6A substitutions (RLG15498; red bar), or an *orf73* gene with a deletion from residue D3 to S58 (RLG15499; green bar). Means and standard deviations from 3 independent assays measuring PFU per milliliter, determined after induction of the *p*_L_ promoter by treatment with mitomycin C, are shown. (C) Schematic diagram of the defective λ prophage genome in the lysogens expressing *orf73* (*orf73+*; RLG15323) or with *orf73* deleted (*orf73*−; RLG15324). These contain deletions of the λ pL operon between the beginning of *c*III and either the beginning of *orf73* or the beginning of *ea22* (deletions indicated with dotted lines), removing λ genes that cause host killing (*kil* and *gam*). The defective prophage also has the rightward phage arm deleted after the fourth amino acid of *cro* (4*aa*Δ*cro*) and contains the heat-inducible *c*I857 lambda repressor. *ybhC* and *bioB* are host genes flanking the prophage insertion site in the bacterial chromosome. (D) Expression of *orf73* from pL in a defective λ lysogen slows cell growth. Strains with the prophage shown in panel C, containing (RLG15323) or lacking (RLG15324) *orf73*, were diluted from cultures grown at 30°C and grown at 42°C to induce pL expression. Optical density was measured every 10 min for ~9 h. Means and ranges of OD_600_ values are from two independent experiments. (E) The growth properties of the defective λ lysogens shown in panel C containing (RLG15323) or lacking (RLG15324) *orf73* are shown. For illustrative purposes, spot titer measurements were made from plates grown at 42°C to induce pL expression, whereas the plating efficiencies (CFU/mL) in the table below the spot titers were determined from plating multiple dilutions of the two strains. After incubation at 42°C, colonies were counted from plates with 30 to 300 colonies. The values shown are averages and ranges from two experiments. (F) Ectopic production of λ Orf73 reduces cell growth rate. Strains with plasmids expressing λ Orf73 (pRLG15593) or TraR (pRLG15592) from the pBAD promoter were grown either in the presence of arabinose (added at time indicated by the arrow) to induce pBAD or in the absence of arabinose. Optical density was measured every 10 min for ~9 h. Means and standard deviations from three independent experiments are shown.

We next examined one of the homologs, λ Orf73, in greater detail *in vivo*. Although bacteriophage λ is perhaps the most intensively studied of all bacteriophages, the functions of some small open reading frames (ORFs) in λ remain unclear (31). Among the genes transcribed from the pL promoter when the phage enters the lytic cycle, there are seven ORFs of unknown function (including Orf73) located between *exo* and *xis* in the left arm of the phage (Fig. 3A). To examine possible roles of λ Orf73 *in vivo*, we examined effects of its expression on phage production and on host cell growth.

In a λ lysogen, the λ pL promoter is repressed by the λ repressor (λ *c*I) (23, 31). Transcription from pL can be induced by inactivating λ repressor, either by treating cells with mitomycin C (inducing an SOS response) or by shifting cells carrying a temperature-sensitive repressor (λ *c*I857) to 42°C. Previous ribosome profiling data indicated that within 20 min of induction of pL and phage entry into the lytic cycle, several of the small open reading frames, including Orf73, are highly expressed, suggesting that they might play a role in phage development (32). Several of the small ORFs were previously implicated in blocking DNA replication initiation of the host DNA and thus termed “*bin*,” but whether the effect of Orf73 on replication was direct or indirect was not addressed (21).

We determined whether Orf73 affects phage yield during a lytic cycle by constructing single-copy λ lysogens that were wild type for *orf73* (*orf73*+) or contained either an in-frame deletion of most of the *orf73* gene (ΔD3–S58 *orf73*) or substitutions for the conserved N-terminal aspartate residues D3 and D6 (Fig. 3A; see Materials and Methods for details of the construction). Cells were treated with mitomycin C to induce lytic phage growth, and phage yields were measured by plating lysates on a λ-sensitive E. coli strain and counting PFU per milliliter (Fig. 3B). The phage yield of a wild-type lysogen was 3- to 4-fold higher than that of lysogens lacking an intact *orf73* gene or containing the D3A and D6A substitutions (Fig. 3B). We conclude that *orf73* is needed for maximum phage yield.

### λ Orf73 affects host growth.

Since the lysogens used for Fig. 3B contained genes in addition to *orf73* that lead to cell killing (e.g., *kil* and *gam*), we used different lysogens to test the specific effects of *orf73* on host growth (Fig. 3C) (21). Wild-type λ Orf73 was expressed from a defective λ prophage unable to produce the transcripts made from the pR promoter and the pR′ promoter (including the head, tail, and lysis genes) and also with deletions of genes in the pL operon between *cIII* and *orf73* (Fig. 3C). The lysogen also contained the gene for the temperature-sensitive λ *c*I857 repressor, allowing induction of pL expression by shifting the temperature to 42°C. We were therefore able to monitor specific effects of λ *orf73* expression on cell growth separate from other phage functions. As a control, we used cells with the same prophage but with a deletion between *cIII* and *ea22*, removing the *orf73* gene (Fig. 3C). After induction of pL at 42°C (*t* = 0), the strains containing or lacking λ Orf73 grew at a similar rate for approximately 1 h, after which the growth of the strain carrying the prophage with wild-type *orf73* slowed appreciably. The strain lacking *orf73* continued growth at the original rate until the strain transitioned to stationary phase (Fig. 3D).

Consistent with the slowed growth observed in the presence of *orf73* in liquid cultures, qualitative spot titer experiments as well as quantitative plating efficiency determinations indicated lower plating efficiency of the strain containing *orf73* (Fig. 3E). The plating efficiencies of the E. coli strain carrying the defective prophage containing the wild-type *orf73* were ~10^4^-fold lower than those of the isogenic strain lacking *orf73* (5.7 × 10^5^ CFU/mL for the strain with *orf73* versus 2.3 × 10^9^ CFU/mL for the strain lacking *orf73*; see the Fig. 3E legend). We conclude that expression of *orf73* slows growth, reducing colony formation.

It was shown previously that TraR expression from an IPTG-inducible promoter on a plasmid slowed cell growth (5). We constructed a plasmid that expressed Orf73 from the arabinose-dependent promoter pBAD to test the effects of *orf73* on host growth in the absence of other phage genes. Soon after induction of either λ Orf73 or TraR from the pBAD vector by addition of arabinose (arrow in Fig. 3F), cell growth slowed appreciably compared to the uninduced cultures. Together, the results shown in Fig. 3 support a model in which expression of *orf73* reduces host growth rate.

### λ Orf73 inhibits host promoters needed for synthesizing the host translation machinery.

**FIG 4:**
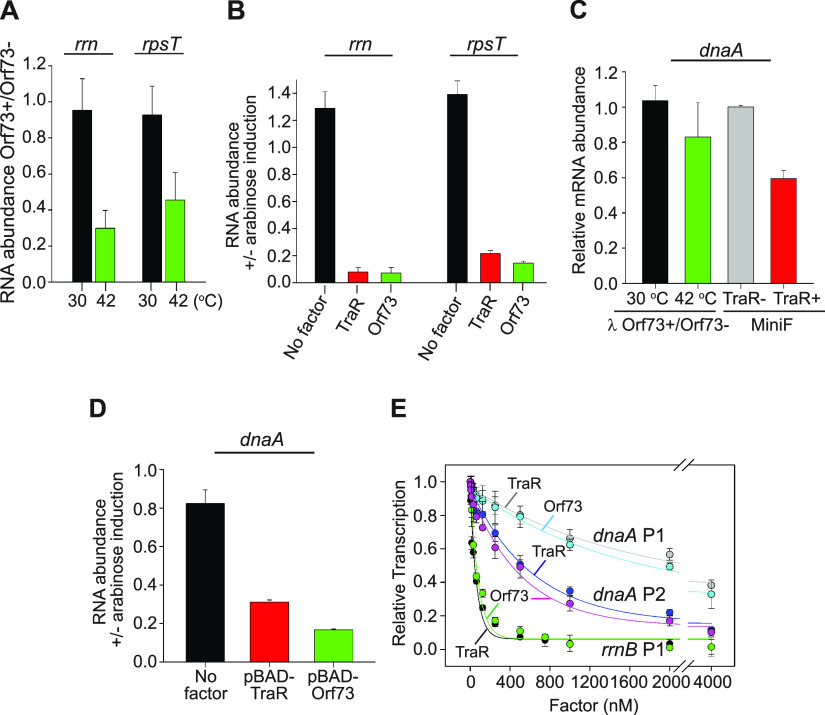
TraR and λ Orf73 strongly inhibit E. coli promoters needed for ribosome synthesis but have only a small effect on *dnaA* promoters. In panels A through D, means and standard deviations from two independent biological replicates, each with three technical replicates, are shown. (A) qPCR analysis of the unstable 16S rRNA precursor (*rrn*) and the *rpsT* mRNA from strains shown in Fig. 3C containing defective λ prophages either with the *orf73* gene (*orf73+*, RLG15323) or without the *orf73* gene (*orf73*−, RLG15324). Cells were grown at either 30°C, where pL is not induced, or at 42°C to induce expression of λ *orf73* from pL. Bars represent the ratio of RNA levels in the *orf73*+ versus *orf73*− strains grown either at 30°C (black bars, no expression of *orf73* from *p*_L_) or at 42°C, at which λ *orf73* was expressed (green bars). See also Materials and Methods. (B) qPCR analysis of the same RNAs as in panel A, except that strains ectopically expressed λ Orf73 (green bar; RLG15593), TraR (red bar; RLG15592), or no factor (black bar; RLG15636) from the pBAD promoter on multicopy plasmids. Values are the ratios of values from cells grown in the presence versus the absence of arabinose for each strain. (C) qPCR analysis of *dnaA* transcripts from E. coli strains carrying the inducible defective λ prophage*s* with or without λ *orf73* shown in Fig. 3C. *dnaA* mRNA levels were expressed as a ratio of values from the *orf73*+ versus the *orf73*− strains grown at either 30°C (control, no *p*_L_ induction; black bar) or at 42°C to induce pL (green bar) as for panel A. For comparison, *dnaA* transcripts were measured in strains with a single-copy mini-F plasmid constitutively expressing TraR (red bar; RLG15333) or a mini-F plasmid without TraR (gray bar; RLG15334). (D) qPCR analysis of *dnaA* transcripts from strains expressing *orf73* (green bar; RLG15593), *traR* (red bar; RLG15592), or no factor (black bar; RLG15636) from the arabinose-induced pBAD promoter on multicopy plasmids as for panel B. Values shown are relative to values for the same strains grown without arabinose induction. (E) λ Orf73 and TraR at high concentrations inhibit transcription from the E. coli *dnaA* promoters *in vitro*. Transcription was measured from a plasmid (pRLG14869) containing the tandem *dnaA* P1 and *dnaA* P2 promoters at concentrations of TraR or λ Orf73 ranging from 4 nM to 4,000 nM. The IC_50_s for inhibition of *dnaA* P2 were similar (~530 nM with TraR or ~420 nM with λ Orf73), and the IC_50_s for inhibition of *dnaA* P1 were likewise similar (~2.2 μM with TraR and ~1.7 μM with λ Orf73). Inhibition of *rrnB* P1 by TraR and λ Orf73 (from Fig. 2B) is shown for comparison. Means and standard deviations are from three independent experiments.

In this section, we present evidence that Orf73 directly inhibits transcription *in vivo* from host promoters responsible for synthesizing the translation apparatus (Fig. 4A). The 16S rRNA is stable, but the region of the rRNA between the 5′ end and the mature 5′ end of the 16S rRNA is processed and attacked by nucleases. Therefore, the synthesis rate of the 16S rRNA was used to infer the synthesis rate of the unstable 5′ leader region of the transcript, as reported previously (33).The pL promoter was induced at 42°C in strains with defective prophages either containing or lacking *orf73* (shown in the schematic in Fig. 3C), and synthesis of the 16S rRNA leader RNA and the unstable r-protein *rpsT* mRNA was measured by reverse transcriptase quantitative PCR (RT-qPCR). Control cultures containing the same two strains were grown at 30°C, where the *p*_L_ promoter was not induced.

When cells were grown at 30°C, the presence/absence of the *orf73* gene on the prophage had little or no effect on production of the rRNA and S20 r-protein mRNA needed for synthesis of the translation apparatus. The ratio of unstable precursor rRNA transcript for the *orf73+* strain relative to the *orf73*− strain was 0.95, and the ratio of the *rpsT* mRNA transcript for the *orf73+* strain relative to the *orf73*− strain was 0.93 (Fig. 4A, black bars). However, when cells were grown at 42°C to induce pL, precursor rRNA synthesis in the strain containing *orf73* was reduced more than 3-fold (to 29% of the value for the strain without *orf73*), and *rpsT* mRNA expression was reduced more than 2-fold (to 45% of the value without *orf73*) (Fig. 4A, green bars). These results are consistent with the observed inhibition of the *rrnB* P1 and *rpsT* P2 promoters by purified Orf73 *in vitro* (Fig. 2).

Effects on host transcription were also determined when λ Orf73 or TraR was expressed ectopically using an arabinose-inducible pBAD promoter fused to the genes for the transcription factors on a multicopy plasmid. Arabinose induction of pBAD on a control plasmid lacking the *orf73* or *traR* genes resulted in very little change in *rrn* (rRNA) or *rpsT* r-protein mRNA synthesis relative to their levels without induction (Fig. 4B, black bars [1.3 ± 0.12 for *rrn* and 1.4 ± 0.13 for *rpsT*]), indicating that arabinose itself had little effect on rRNA or *rpsT* expression. In contrast, expression of TraR and λ Orf73 by induction of the pBAD promoters reduced rRNA promoter activity ~12-fold (0.08 ± 0.04) and 14-fold (0.07 ± 0.005), respectively, relative to their levels without induction. *rpsT* P2 promoter activity was reduced ~5-fold by TraR (0.21 ± 0.009) and ~7-fold by λ Orf73 (0.14 ± 0.001) (red and green bars, respectively, in Fig. 4B).

Inhibition of *rrn* or *rpsT* P2 activity by λ Orf73 was greater when Orf73 was expressed from the multicopy pBAD plasmid than from the single-copy prophage, likely reflecting differences in the levels of Orf73 expression (compare Fig. 4A and B). Together, the inhibition of *rrn* and *rpsT* expression by Orf73 *in vivo* (Fig. 4A and B) is consistent with the inhibition of the *rrnB* P1 and *rpsT* P2 promoters *in vitro* (Fig. 2B and Fig. S3) and suggests that the effects of Orf73 on transcription *in vivo* are direct. The strong inhibition of rRNA and r-protein mRNA synthesis after ectopic TraR expression (Fig. 4B) is also consistent with the previously reported effects of expression of TraR on the activities of *rrnB* P1 promoter*-lacZ* fusions (5).

### Examination of the effect of λ Orf73 on transcription from the *dnaA* promoter.

It was noted previously that continuous expression of the pL operon caused arrest of initiation of new rounds of host DNA replication. λ Orf73 was implicated in this inhibition, although the mechanistic details were unclear (21). It was proposed recently that ppGpp inhibits synthesis of DnaA, the initiator protein for DNA replication (34). Since Orf73 and TraR are mimics of ppGpp/DksA, we used qPCR to measure their effects on *dnaA* mRNA production. λ Orf73 was expressed from the defective prophage illustrated schematically in Fig. 3C, whereas TraR was expressed from a mini-F plasmid. Although there was a small effect (~40% decrease) on *dnaA* expression when TraR expression was induced from the mini-F plasmid (Fig. 4C, compare gray versus red bars), *orf73* expression from a prophage had little or no effect on *dnaA* mRNA levels (Fig. 4C; black and green bars are within error). Inhibition of *dnaA* expression was greater when either λ Orf73 or TraR was produced by induction of the pBAD promoter on a high-copy-number plasmid (Fig. 4D), but the extent of inhibition of *dnaA* transcription was still only about one-half to one-third as large as that of rRNA expression under similar conditions (compare Fig. 4D and B).

To examine the effects of λ Orf73 and TraR on *dnaA* transcription more quantitatively in the absence of other confounding variables, we measured their effects at a range of protein concentrations *in vitro*. The *dnaA* gene has two promoters, *dnaA* P1 and *dnaA* P2. The *dnaA* P2 promoter has been reported to be ~3-fold stronger than P1 (35, 36). We found that TraR and λ Orf73 directly inhibited *dnaA* P2, but the IC_50_s for inhibition of this promoter were high, ~530 nM and ~420 nM for inhibition by TraR and Orf73, respectively (Fig. 4E). TraR and λ Orf73 also inhibited *dnaA* P1, but with an even higher IC_50_, 1 to 2 μM (Fig. 4E). For comparison, the effects of TraR and Orf73 on *rrnB* P1 (IC_50_, 50 nM for each) are shown on the same graph. Thus, the concentrations of λ Orf73 and TraR required for inhibition of the *dnaA* promoters *in vitro* were ~10- to 40-fold higher than for inhibiting *rrnB* P1 (Fig. 4E). These results are consistent with the smaller effects of λ Orf73 and TraR on transcription from *dnaA* that we observed *in vivo* when TraR and λ Orf73 were produced from single-copy or multicopy constructs. We conclude that the effects of λ Orf73 and TraR on *dnaA* transcription, even if significant, are much smaller than on *rrnB* P1 transcription *in vivo*.

### TraR significantly increases transcription *in vitro* from F element promoters required for conjugation, but λ Orf73 does not increase transcription from lambda lytic promoters.

**FIG 5:**
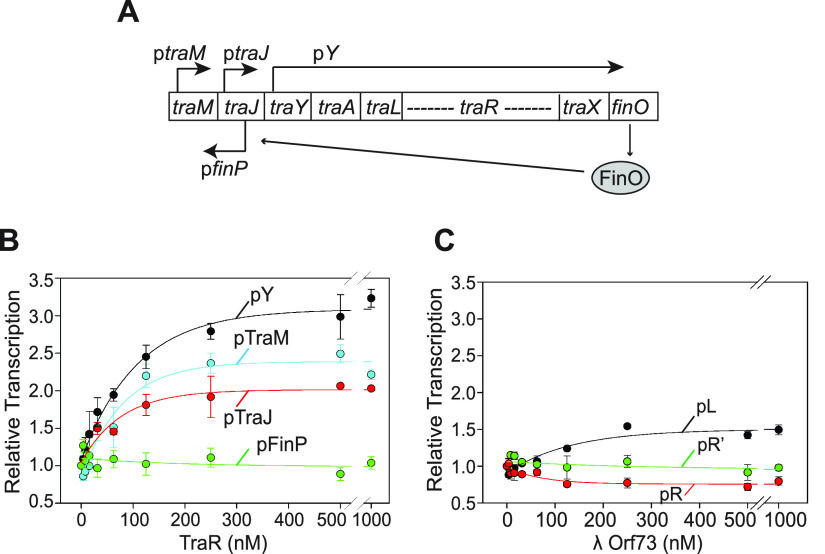
E. coli TraR stimulates transcription from F element *tra* operon promoters, whereas λ Orf73 has much smaller effects on λ lytic promoters. (A) Map of the ~30-kb *tra* operon showing the major promoters p*traM*, p*traJ*, p*finP*, and p*Y* (adapted from references 37–39). The TraR gene, transcribed from the p*Y* promoter, is shown, but for brevity, most other ORFs in the *traY* operon are not shown. FinO and the antisense RNA FinP prevent expression of TraJ, which is an activator of the p*Y* promoter. (B) Multiround *in vitro* transcription from p*traM* (pRLG15296), p*traJ* (pRLG15297), p*Y* (pRLG15298), or p*finP* (pRLG15374) without TraR or with TraR concentrations from 4 nM to 1,000 nM. (C) Multiround *in vitro* transcription from the λ pL (RLG15510), pR (RLG15512), and pR′ (RLG15513) promoters without λ Orf73 or with 4 nM to 1,000 nM λ Orf73. In panels B and C, averages and standard deviations from three independent experiments are shown.

TraR and λ Orf73 strongly inhibited host promoters that play critical roles in ribosome synthesis, likely explaining their effects on host growth. We next tested whether TraR and λ Orf73 might also affect promoters on their own extrachromosomal elements. We cloned 4 promoters from the *tra* operon (p*Y*, p*traJ*, p*traM*, and p*finP* (37–39) (Fig. 5A)), and we tested their responses to TraR *in vitro* (Fig. 5B). TraR directly increased transcription from 3 of the 4 promoters (p*Y* by ~3.2-fold, p*traM* by ~2.5-fold, and p*traJ* by ~2-fold), suggesting that TraR might increase the efficiency of conjugation not only by strongly inhibiting host transcription but also by increasing transcription of the *tra* operon itself. The *finP* promoter, which codes for a negative regulator of the *tra* operon, was unaffected by concentrations of TraR even as high as 4 μM, in support of the model that TraR activates only *tra* promoters that promote conjugation.

To determine whether λ Orf73 increases lytic growth not only by inhibiting host promoters that would compete with λ promoters for RNAP but also by directly activating λ promoters required for λ lytic functions, we cloned and tested effects of λ Orf73 on the 3 major lytic promoters *in vitro*, pR, pR′, and pL. Only pL increased at all, and the increase was only 1.4-fold (Fig. 5C), suggesting that effects of λ Orf73 on phage burst size (Fig. 2C) are primarily indirect from inhibition of host promoters.

## DISCUSSION

Prior to the work reported here, the physiological roles of TraR homologs were unknown. We show here that five of bacteriophage-encoded TraR homologs function similarly to TraR, albeit not identically, by binding to RNA polymerase and regulating specific promoters. The widespread conservation of the TraR homolog family in proteobacterial phages and conjugative plasmids suggests that they play significant roles in phage production and DNA transfer, but there are enough sequence differences among the factors to accommodate phage- or plasmid-specific roles. Since TraR is not absolutely required for conjugation by F (37, 38, 40), and λ Orf73 is not absolutely required for lytic growth (Fig. 3), we suggest that these factors play accessory rather than essential roles in the biology of the extrachromosomal elements. We suggest that bacteriophages utilize TraR homologs rather than ppGpp/DksA in order to reprogram host transcription without inhibiting host enzymes needed for phage development.

### Conservation of residues in the TraR homologs that are important for function.

As described in Results, the five TraR homologs contain conserved residues that were shown previously to be important for regulation of transcription by TraR, and structural features like those in TraR are predicted to be present in the homologs (Fig. S2). In the N-terminal helix, the homologs contain the conserved DxxDxA motif, which interacts with the active-site region of RNAP. These and other residues form a network of interactions with RNAP that result in large conformational changes of RNAP that inhibit transcription initiation (17). Substitutions for these residues in λ Orf73 reduce its activity, as was shown previously for TraR (Fig. 2E and 3B; and Fig. S3) (6). Lack of full conservation of this motif in two of the homologs, Phage P2 Orf82 and *X. bovienii* Gp34, could potentially contribute to the lower activities of these proteins in our *in vitro* assays (Fig. 2B).

We note that there is a similar conserved motif (DxxExA) in the coiled-coil tip of the structurally similar transcription factors GreB and GreA. However, TraR and DksA regulate different steps in the transcription cycle than the Gre factors, and their coiled-coil tip regions interact with different conformations of the highly mobile active-site region of RNAP. The TraR/DksA coiled-coil tip region interacts with RNAP holoenzyme before promoter binding as part of a complex network of TraR/DksA interactions with RNAP that alter the kinetics of open complex formation prior to catalysis (17–19). In contrast, the GreB tip region interacts with RNA in the active-site region of backtracked elongation complexes, and it is proposed to stimulate the intrinsic RNA cleavage activity of RNAP by stabilizing Mg^2+^ binding (41).

In the globular domain, the homologs each contain residues analogous to R48, shown previously to be important for TraR binding to the “rim helices” at the entrance to the RNAP secondary channel, and R49, which is required for interaction with a lineage specific insertion found in the β′ subunit in proteobacteria, β′Si3 (6, 17). This interaction with β′Si3 contributes to a large reorientation of the β′Si3 domain in the TraR-RNAP-promoter complex and plays a role in activation of transcription by TraR (6, 17).

The TraR homologs also contain residues corresponding to the C-terminal helix of TraR, a region that interacts with another lineage-specific insertion in proteobacterial RNAP, the β subunit Si1 insertion. The TraR interaction with βSi1 causes a large rotation of the β lobe and alters the conformation of the promoter DNA binding surface in the main channel of the enzyme (17). An RNAP variant lacking βSi1 does not support TraR-mediated regulation of transcription (6).

The presence of these conserved sequences in the TraR homologs suggests that the interactions with the lineage-specific sequences in RNAP also play a role in their function as transcriptional regulators. The importance of the βSi1 and β′Si3 insertions in the mechanism by which TraR regulates transcription (6) is consistent with the finding that TraR homologs are found primarily in the bacterial phylum that contains these lineage specific insertions, the proteobacteria (17, 42, 43).

### Potential roles of TraR in F element transfer.

Previous work has focused on two nonexclusive models for accessory roles of TraR, both of which rely on its ability to regulate transcription initiation. TraR is expressed as part of the large transfer (*tra*) operon, and it enhances Eσ^E^ activity both directly and indirectly (16). One model is that the products encoded by the TraR-induced transcripts might play a role in alleviating the disruption of the integrity of the outer membrane proteins (OMPs) and periplasm during assembly of the pilus during mating (16).

A second model is based on the observation that TraR inhibits rRNA and r-protein synthesis and can stop cellular growth when expressed ectopically, even in single copy (Fig. 3F and 4B; Fig. S3) (5, 6). Transcription of rRNA and r-protein mRNAs represents a large fraction of total transcription during exponential growth in rich medium, the condition under which conjugation is most efficient (15, 38). Inhibition of ribosome synthesis and cell growth in donor and recipient cells would increase the amount of Eσ^70^ RNAP available for transcription of genes needed for conjugation and/or increase the amount of core RNAP available for formation of other forms of RNAP holoenzyme, such as Eσ^E^.

We show here that TraR modestly inhibited *dnaA* transcription when it was expressed from a single copy mini-F element (pOX38 *traR* MiniF) (Fig. 4C), and it had a larger effect when induced from the pBAD promoter on a multicopy plasmid (Fig. 4D). We suggest that TraR might temporarily reduce initiation of host DNA replication and cell division to prevent partial segregation of the plasmid into daughter cells.

Finally, we found that TraR directly increases the activities of 3 promoters in the F element, including p*Y*, the main promoter for the *traY* operon, p*traJ*, a promoter for expression of TraJ, an activator of p*Y*, and p*traM*, a promoter for expression of TraM, a protein that binds to the origin of transfer, *oriT* (Fig. 5A and B). We suggest that TraR might improve the efficiency of conjugation by increasing expression of pilus biosynthesis, other mating-related genes, and its own transcription. In summary, TraR may optimize the efficiency of the DNA transfer process in several different ways, although we note that an effect of a *traR* mutation on conjugation efficiency has yet to be demonstrated directly.

### Role of λ Orf73 in lambda phage production.

The five bacteriophage-encoded TraR homologs studied here all affect transcription initiation, but the physiological role of this regulation differs from that by TraR, since phages and conjugative plasmids have different “lifestyles.” λ Orf73 (referred to as Orf2310 in reference 32) is expressed from the pL promoter early in lytic phase (5 to 20 min after induction) (32), along with gene products needed for transcription antitermination (λ N), recombination (Exo and Gam), and the lysis-lysogeny decision (*c*III). Consistent with its expression from pL during the lytic phase, λ Orf73 increased phage yield ~4-fold following induction of a lysogen (Fig. 3B). Previous reports proposed that other short open reading frames in the pL operon *exo-xis* region (Fig. 3A) play a role in the lysis-lysogeny decision (44, 45), but the specific functions of those gene products are not known.

Expression of λ Orf73 from its native promoter or from a multicopy plasmid affected host gene expression (e.g., transcription from rRNA and r-protein promoters), cell growth, and colony formation of the host strain (Fig. 3D to F and 4A and B). λ Orf73 was as efficient as TraR in inhibition of stable RNA and r-protein promoters both *in vitro* (Fig. 2B; Fig. S3) and *in vivo* (Fig. 4A and B). However, λ Orf73 did not affect expression from the λ lytic promoters that we tested (Fig. 5C). We suggest that the major effects of λ Orf73 on phage production derive from its effects on host promoters, redirecting the cell’s transcriptional resources away from production of the translation machinery. Existing ribosome pools in the exponentially growing cells in which phage production is optimal would be sufficient to translate phage proteins, and new ribosome synthesis would not be needed for a cell destined for lysis.

Previous work suggested a role for *λ orf73* and three other adjoining phage genes (*ea22*, *ea8.5*, and *orf55*) in the inhibition of host DNA replication initiation (21). In contrast with its effects on rRNA transcription when expressed from a nonreplicating defective prophage (Fig. 4A), λ Orf73 reduced levels of *dnaA* mRNA weakly if at all (Fig. 4C). However, it is possible that the concentration of λ Orf73 would be high enough during a lytic cycle to reduce DnaA synthesis significantly, since the number of phage genomes per cell would be much higher under these conditions. Alternatively, since λ Orf73 inhibits rRNA transcription, which in turn affects local DNA supercoiling, the observed effect of the cluster of short open reading frames (including λ Orf73) on replication initiation could be mediated by changes in supercoiling of the origin region, as has been proposed for effects of ppGpp/DksA on replication initiation (46). More complex explanations for the effects of ppGpp on replication initiation unrelated to either supercoiling status or *dnaA* transcription initiation have also been proposed (47), although it is not clear that these would explain the effect of λ Orf73 on lytic growth.

We also note that new DnaA synthesis is essential for initiation of DNA synthesis (48). Newly synthesized DnaA preferentially binds ATP because of the high ratio of ATP to ADP in the cell, and the ATP-bound form of DnaA (the active form) is a relatively small proportion of the total DnaA pool. Therefore, we suggest that a small decrease in *dnaA* transcription by λ Orf73 (or TraR; see above) and a corresponding reduction in the concentration of newly synthesized DnaA could disproportionally reduce replication initiation.

In contrast with TraR, which activated *tra* operon promoters as well as certain host promoters, λ Orf73 activated the three host promoters we tested weakly if at all (*thrABC*, *argI*, and *rpoH* P3) (Fig. 2F). The significance of the failure of λ Orf73 to activate host promoters *in vitro* is unclear because of the small number of promoters tested. λ Orf73 could be mechanistically incapable of activating transcription, since it has 2 extra cysteine residues for a total of 6, compared to the 4 in TraR and the other TraR homologs. Four of the cysteines in λ Orf73 correspond to those that form the C4 type zinc finger in TraR (the CxxC-17nt-CxxC motif). The two others are the residues corresponding to TraR positions 38 and 55 (Fig. 1A and Fig. S2). The presence of the additional cysteines in λ Orf73 could potentially affect the folding of the globular domain, and therefore its interaction with β′Si3. Altered folding of the λ Orf73 globular domain could account for its inability to activate transcription, since the TraR globular domain interaction with β′Si3 plays a significant role in the activation mechanism (17). Consistent with the hypothesis that the extra cysteines affect folding, the protein in the RaptorX model for wild-type λ Orf73 is largely unstructured, but the models for λ Orf73 variants with substitutions in one or both of the two extra cysteines have features similar to those of TraR and the other TraR homologs (Fig. S2).

Our conclusion that λ Orf73 expression increases phage yield differs from that in a previous paper (22) in which it was reported that λ Orf73 reduced phage yield during lytic growth. We suggest that this difference may reflect the different methods of inactivating the *orf73* gene and/or different methods for inducing lytic growth (by induction of an SOS response in a lysogen in our experiments versus outside infection of the host strain in the previous study (22)). We note that λ *orf73* was inactivated by point mutations or by an in-frame deletion in our experiments (Fig. 3A), changes that should not have caused polar effects on adjacent downstream genes.

### Potential roles of the other four TraR homologs.

Like λ Orf73, three of the other phage-encoded TraR homologs tested here (P2 Orf 82, VP882 Orf 61, and VHML Orf 8) are encoded by regions of the phage genomes with predicted functions related to the lysis-lysogeny decision and regulation of transcription or DNA replication and other small ORFs of unknown function (24–26). However, the identities of the genes and their organization in these regions are not highly conserved, likely reflecting unique adaptations of the phages to their respective hosts. For example, the gene for the TraR homolog Orf61 is located in a region of the *Vibrio* VP882 phage genome that contains genes involved in a quorum sensing mechanism regulating phage induction by monitoring host cell density (26, 49). It is not known whether Orf61 plays a role in the quorum sensing mechanism. The TraR homolog from *X. bovienii*, Gp34, is predicted to be encoded by a phage gene, but to our knowledge, no information is available about its context and expression.

We suggest that, as described above for λ Orf73, the TraR homologs in the other phages inhibit host rRNA and r-protein transcription to enable use of the host RNA polymerase for phage production during the lytic cycle. In support of this proposal, we found that VP882 Orf61 directly inhibits transcription at *Vibrio rrnA/B* P1 promoters and the *Vibrio rpsT* P2 promoter using *Vibrio* RNAP (Fig. 2D), consistent with a role for Orf61 in regulating transcription initiation in its native host. Also consistent with a role for the TraR homologs in regulating host gene expression, overexpression of P2 Orf82 was reported previously to stop cell growth (50). In its native context, P2 Orf82 is expressed from the Pe (early) promoter in an operon that also contains genes for DNA replication and several small ORFs of unknown function, consistent with the idea that Orf82 plays a role during the lytic cycle (24). It is possible that some of the other TraR homologs could regulate promoters on the phages that encode them, although λ Orf73 did not affect the lambda promoters that we tested (Fig. 5).

### Other TraR/DksA family homologs.

The TraR homologs that we investigated here are representative of a large group of proteins of similar length to TraR (73 residues), but we note that proteins of widely varying length and unknown function are classified as TraR/DksA homologs based primarily on the presence of a C4 Zn^2+^ finger motif.

Some TraR homologs that contain a conserved C4 Zn^2+^ finger are longer than TraR, either intermediate in size between TraR (73 residues) and DksA (151 residues), similar in length to DksA, or even longer than DksA. A few proteins in the intermediate length category do not function like DksA or TraR, e.g., the 105-residue RSP0166 protein from Rhodobacter sphaeroides (51) and the 114-residue SMc00049 protein from Sinorhizobium meliloti (52). These proteins appear to lack several critical features of DksA, including the conserved DxxDxA motif, the ppGpp-interacting residues, and the C-terminal helix (10, 11). Thus, their lack of function is not surprising. Some other DksA homologs lack a C4 Zn^2+^ finger, but they contain other conserved functionally important features and function like DksA (e.g., R. sphaeroides RSP2654 [51, 53] and S. meliloti SMc00469 [52]). The Pseudomonas aeruginosa DksA2 protein functions like DksA, and its X-ray structure indicated that it has the same globular domain fold as DksA even though it lacks a zinc finger (54). Thus, a C4 Zn^2+^ finger is not always required for DksA-like function, and not all C4 Zn^2+^ finger proteins function like DksA. We have not yet identified TraR homologs that lack the C4 Zn^2+^ finger.

There are other small secondary channel binding proteins that do not contain the C4 Zn^2+^ finger motif and are not homologs of TraR or DksA but are distant homologs of the E. coli transcription elongation regulators GreA and GreB. Rnk, a protein in this class, does not carry out Gre functions, but it does bind to the secondary channel and competes with the Gre factors (and DksA) for binding to RNAP (55).

### The TraR homologs have a different role from ppGpp/DksA even though they share mechanistic properties.

TraR and λ Orf73 have effects on RNAP like ppGpp and DksA together. Unlike TraR and its homologs, which are encoded by extrachromosomal elements and are produced only at specific times and under specific conditions, DksA is maintained at fairly constant levels throughout growth (56). However, in contrast to TraR and its homologs, which function independently of ppGpp, DksA requires ppGpp binding to the DksA/RNAP complex (10). Although DksA can inhibit transcription initiation in the absence of ppGpp *in vitro*, this is observed only at very high DksA concentrations when standard multiround assays are used. Regulation *in vivo* occurs only in the presence of both DksA and ppGpp (10, 57). We suggest that TraR and its homologs have evolved to regulate transcription when phage growth and conjugation are most efficient, i.e., at high growth rates in rich medium (15, 38), conditions under which ppGpp levels are low and thus DksA is much less active.

ppGpp levels are increased by nutritional limitation. In addition to binding to RNAP, ppGpp also binds directly to 50 or more cellular proteins during a stringent response in E. coli, inhibiting ribonucleotide synthesis, assembly of ribosomes, initiation and elongation of translation, and a number of metabolic pathways (8, 9). Inhibiting these functions would likely be detrimental to phage production and conjugation. Thus, we suggest that TraR and its homologs have evolved to function independently of ppGpp so as not to cause the disruptions to metabolism and translation caused by ppGpp binding to targets other than RNAP. In summary, TraR and its homologs carry out only a subset of the functions performed by DksA and ppGpp during a stringent response, increasing the availability of RNAP for phage and F element transcription without disrupting the host machinery needed for phage and F element synthetic functions.

## MATERIALS AND METHODS

### Strains, plasmids, oligonucleotides, and Geneblock sequences.

Strains and plasmids are listed in Table S1, oligonucleotide sequences in Table S2, and Geneblocks in Table S3. Bacteria were grown in LB Lennox medium, on LB agar plates, or on M9 glucose minimal medium agar plates, as indicated in the text. The medium was supplemented with ampicillin (100 μg/mL) or kanamycin (30 μg/mL) when needed. IPTG (1 mM) or arabinose (0.2%, wt/vol) was used to induce expression of the factors from the pTrc, pBAD, and pET vectors as appropriate. Plates were incubated at 30, 37, or 42°C as indicated in the figure legends. Plasmid construction for transcription factor expression has been described (6). Antibiotic resistance, PCR, DNA sequencing, and/or phenotypic assays were used to verify the constructs.

10.1128/mbio.00952-22.5 TABLE S1 Strains and plasmids used in this study. Download Table S1, DOCX file, 0.02 MB. Copyright © 2022 Gopalkrishnan et al. 2022 Gopalkrishnan et al. https://creativecommons.org/licenses/by/4.0/ This content is distributed under the terms of the Creative Commons Attribution 4.0 International license.

10.1128/mbio.00952-22.6 TABLE S2 Oligonucleotides used in this study for plasmid construction, DNA sequencing, and qPCR and for creating mutations. F, forward primer; R, reverse primer. Download Table S2, DOCX file, 0.01 MB. Copyright © 2022 Gopalkrishnan et al. 2022 Gopalkrishnan et al. https://creativecommons.org/licenses/by/4.0/ This content is distributed under the terms of the Creative Commons Attribution 4.0 International license.

10.1128/mbio.00952-22.7 TABLE S3 Oligonucleotides used in this study for construction of λ phage variants. Download Table S3, DOCX file, 0.01 MB. Copyright © 2022 Gopalkrishnan et al. 2022 Gopalkrishnan et al. https://creativecommons.org/licenses/by/4.0/ This content is distributed under the terms of the Creative Commons Attribution 4.0 International license.

### Structural modeling of TraR homologs.

Models were generated by RaptorX Protein Structure Prediction (19) (http://raptorx.uchicago.edu/StructurePrediction/), using the RaptorX web server-defined best structural templates (either PDB 5W1SM [TraR] or PDB 1TJLA [DksA]). See also the Fig. S2 legend.

### Purification of TraR, TraR homologs, and DksA.

The genes encoding TraR and the TraR homologs from phages λ (Orf73), P2 (Orf82), and VP882 (Orf61) were cloned into pET28a-His_10_-SUMO, transformed into E. coli BL21(DE3) *dksA*::Tn*10*, and purified via nickel-nitrilotriacetic acid (Ni-NTA) chromatography (Qiagen), as described elsewhere (17). The N-terminal His_10_-SUMO tag was cleaved off with Ulp1 protease. The purified, tagless protein was stored in buffer containing 10 mM Tris-HCl (pH 8.0), 0.1 mM EDTA, 2 mM dithiothreitol (DTT), 250 mM NaCl, and 50% glycerol. TraR homologs from *X. bovienii* Gp34 and phage VHML Orf8 were purified from a E. coli BL21(DE3) *dksA*::Tn*10* strain carrying the pET28a-homolog-His_6_ plasmids and purified by Ni-NTA chromatography essentially as described elsewhere (6). The C-terminal His_6_ tag was cleaved off with thrombin, leaving 4 amino acids (LVPR) at the C-terminal end. The tagless version of E. coli TraR and TraR with the 4-residue tag had the same activities (6, 17).

### E. coli RNAP and V. cholerae RNAP.

Native E. coli and Vibrio cholerae RNAP holoenzymes were purified as described elsewhere (58, 59). Both *Vibrio* TraR homologs studied here derive from phages that naturally infect V. cholerae as well as other *Vibrio* species. Because the V. cholerae native holoenzyme was slightly subsaturated for σ^70^, 2× additional σ^70^ was added to the holoenzyme before use in transcription experiments. V. cholerae σ^70^ was purified from inclusion bodies. His-tagged Vibrio cholerae σ^70^ was overexpressed from pET21a in T7 Express cells (New England Biolabs [NEB]). Following solubilization of inclusion bodies as described in reference 60, His-tagged σ^70^ was bound to a 5-mL HisTrap column, refolded in 10 mM Tris-HCl (pH 7.6), 500 mM NaCl, and 10 mM imidazole, and then eluted with a gradient of 10 to 200 mM imidazole. σ^70^-containing fractions were pooled and concentrated using a Vivaspin column (Sartorius) to 0.4 mg/mL, before dialysis in storage buffer containing 10 mM Tris-HCl (pH 7.6), 150 mM NaCl, 10 mM MgCl_2_, 0.1 M KCl, 1 mM DTT, and 50% (vol/vol) glycerol.

### Iron cleavage competition assay for TraR homolog binding to RNAP.

The binding activity of TraR or the TraR homologs for E. coli RNAP was determined by competition with ^32^P-DksA, prebound to a molar excess of RNAP containing Fe^2+^ instead of Mg^2+^ in the active site. Hydroxyl radicals generated at the RNAP active site by the addition of DTT result in cleavage of the DksA that is bound to RNAP, producing fragments of ^32^P-DksA that can be separated from intact DksA by electrophoresis on a 4 to 12% SDS gel. The fraction of DksA cleaved in the presence of TraR or TraR homolog competitor, relative to the absence of competitor, was determined by phosphorimaging (see reference 6 for details).

### Construction of λ lysogens.

A λ lysogen of E. coli MG1655 (XTL850) was constructed and shown to contain only a single copy of the prophage (61). XTL850 derivatives with wild-type and mutant λ *orf73* alleles (Fig. 3A) were constructed by λ Red recombineering as described previously (62). An insertion of *tet-sacB* in λ *orf73* was constructed in strain XTL850 with selection for tetracycline resistance, using plasmid pKM208 containing λ Red functions (63) and the PCR product T-SACK, produced from oligonucleotides XT1028 and XT1029 (64). Derivatives of the resulting strain (XTL1055) containing λ *orf73* D3A, D6A (XTL1071 = RLG15498), λ *orf73* ΔD3–S58 (XTL1072 = RLG15499), or wild-type (WT) λ *orf73* (XTL1073 = RLG15500) were constructed by replacement of λ *orf73 tet-sacB* using oligonucleotide XT1032, XT1033, or XT1043, respectively, with selection on sucrose agar and subsequent removal of plasmid pKM208 by growth in LB and screening of single colonies for sensitivity to ampicillin.

Strains with a defective λ prophage containing or lacking λ *orf73* (Fig. 3C) were constructed from strain HME6 (65). An HME6 derivative with an insertion of *cat-sacB* in the λ *gam* gene (XTL241) was constructed using PCR product CC4231 produced from primers XT409 and XT410 (64), with selection for chloramphenicol resistance. A derivative of XL241 with the λ *c*III promoter fused to *orf73* was constructed by deletion of the DNA sequences between λ *c*III and *orf73* using oligonucleotide XT415 with selection on sucrose agar to produce strain XTL255 (= RLG15323). A derivative of XTL241 with the λ *c*III promoter fused to λ *ea22*, thereby deleting λ *orf73* (Fig. 3C), was constructed by deletion of the DNA sequences between *c*III and λ *ea22* using oligonucleotide XT419, with selection on sucrose agar to produce strain XTL256 (= RLG15324).

### Growth curves.

For experiments whose results are shown in Fig. 3D and F, cultures were grown overnight in LB at 37°C and subcultured in triplicate starting at an optical density at 600 nm (OD_600_) of 0.02 in 96-well plates in a plate reader either at 37°C (for Fig. 3F) or 42°C (for Fig. 3D). OD_600_ measurements were taken every 10 min for ~10 h.

### Plating efficiency measurements.

For the experiment whose results are shown in Fig. 3E, duplicate overnight cultures of E. coli strains RLG15323 (*orf73*+) and 15324 (*orf73*−) were grown in LB at 30°C, and serial dilutions (10^−1^ to 10^−9^) were prepared in LB from each culture. Ten-microliter aliquots were spotted onto LB plates, and plates were incubated at 42°C overnight.

### Bacteriophage λ phage production assays (PFU/mL).

For the experiment whose results are shown in Fig. 3B, isogenic lysogenic strains RLG15500 (λ *orf73* wild type), RLG15498 (λ *orf73* D3A, D6A), and RLG15499 (λ *orf73* ΔD3–S58) were grown to an OD_600_ of 0.2 and were then treated with mitomycin C (1 μg/mL), and growth was continued for 3 h. Mitomycin C induces DNA damage, which activates RecA and promotes the autocleavage of the λ *c*I repressor, thereby inducing lytic growth. The cells were then pelleted, and the cleared lysate containing the released phage particles was titrated. Dilutions of the cleared lysate were adsorbed to RLG14475 (MG1655) by incubation without shaking for 30 min at 37°C. Cells were then mixed gently with melted soft agar at 50°C, poured evenly on dry, prewarmed LB plates, and incubated overnight. PFU were measured using plates containing fewer than 300 plaques (average of triplicate plates).

### *In vitro* transcription.

Multiple-round *in vitro* transcription reactions were carried out at 22°C in a buffer containing 170 mM NaCl as described elsewhere (6). TraR, TraR homologs, DksA, and/or ppGpp were added at the concentrations indicated in the figures and figure legends.

### Site-directed mutagenesis.

Substitutions were introduced into a pET8a-SUMO-His_10_ vector using the QuikChange Lightning Multi site-directed mutagenesis kit (Agilent) and the mutagenic oligonucleotides listed in Table S2. Mutations were confirmed by DNA sequencing.

### Western blotting.

Western blotting was performed using standard procedures using a rabbit anti-TraR polyclonal antibody (Covance Research) (Fig. S4). RLG15592 (MG1655 pBAD-TraR) was inoculated in LB at a starting OD_600_ of 0.025 and grown at 37°C. When strains reached an OD_600_ of 0.15 to 0.2, arabinose (0.2% final concentration) was added to induce factor expression, and samples were collected at the intervals indicated in the figure (6).

10.1128/mbio.00952-22.4 FIG S4 TraR levels after arabinose induction from the pBAD promoter on plasmid pRLG15592. TraR levels were analyzed using Western blots with anti-TraR polyclonal antibody after induction from pBAD with 0.2% arabinose. Lysate was loaded at 0.5 μg per lane. Arabinose was added to cells at an OD_600_ of 0.15. TraR levels peaked at OD_600_ of 0.6. Download FIG S4, EPS file, 0.9 MB. Copyright © 2022 Gopalkrishnan et al. 2022 Gopalkrishnan et al. https://creativecommons.org/licenses/by/4.0/ This content is distributed under the terms of the Creative Commons Attribution 4.0 International license.

### RNA extraction and RT-qPCR analysis.

Strains harboring plasmids containing pBAD fusions were grown with shaking at 37°C in LB medium to early log phase (OD_600_ = 0.2), arabinose was added, cells were harvested when strains reached an OD_600_ of 0.6 (when the accumulated factor was at maximum concentration [Fig. S3]), and RNA was extracted. Cells were harvested and RNA was extracted at an OD_600_ of 0.6 for strains carrying mini-F plasmids expressing TraR. For strains carrying λ prophages encoding the temperature-sensitive *c*I857 repressor, strains were subcultured at an OD_600_ of 0.025 from overnight cultures grown at 30°C and switched to 42°C at an OD_600_ of 0.2 to induce the *p*_L_ promoter, and RNA was harvested when strains reached an OD_600_ of 0.6. For each RT-qPCR measurement, RNA samples were extracted from two independent biological replicates. Each cell sample was mixed with ice-cold ethanol-phenol stop solution to inactivate cellular RNases and centrifuged, and RNA was purified using the Direct-zol RNA MicroPrep protocol (Zymo Research). RNA integrity (16S and 23S rRNA) was verified by agarose gel electrophoresis.

RNA was treated with DNase I (NEB), and total RNA (0.5 μg) was used as a template for cDNA synthesis using the iScript cDNA synthesis kit (Bio-Rad). RT-qPCR was performed with iTaq universal SYBR green supermix (Bio-Rad) on a CFX Connect real-time PCR detection system (Bio-Rad) using the cDNA as a template. Transcripts were amplified using a 0.5 μM concentration of the oligonucleotides listed in Table S2 to generate a 90- to 120-nucleotide (nt) product that was verified by melting curve analysis. A 300-nt product generated from the 3′ end of the 16S rRNA was used to determine the amount of RNA added. Control reactions for each target were carried out in the absence of a cDNA template. The fold change in expression of the target gene relative to the 16S rRNA internal control was calculated with the 2^−ΔΔ^*^CT^* equation using CFX Manager software, as described previously (12, 66). Results shown are averages and standard deviations for two independent biological replicates, each consisting of three technical repeats.

10.1128/mbio.00952-22.8 TABLE S4 Geneblocks used in this study. Start and stop codons, promoter elements, and transcription start sites are in bold and underlined. Download Table S4, DOCX file, 0.02 MB. Copyright © 2022 Gopalkrishnan et al. 2022 Gopalkrishnan et al. https://creativecommons.org/licenses/by/4.0/ This content is distributed under the terms of the Creative Commons Attribution 4.0 International license.
