# Supplementary material for: Homologs of the Escherichia coli F Element Protein TraR, Including Phage Lambda Orf73, Directly Reprogram Host Transcription
Source: mBio. 2022 May 18;13(3):e00952-22. doi: 10.1128/mbio.00952-22 (PMC9239242; doi:10.1128/mbio.00952-22)
Supplement: TABLE S1 [file mbio.00952-22-s0005.docx]

**Table S1: Strains and Plasmids**

| **Strain Number** | **Genotype** | **Source** |
| --- | --- | --- |
| RLG4677 | BL21DE3 | Novagen |
| RLG6348 | *dksA::tet* VH1000 = MG1655 *pyrE*+ *lacI lacZ* | (1) |
| RLG7075 | BL21DE3 Δ*dksA* | (1) |
| RLG9933 | MG1655 (*ilvG rfb-50 rph-1*) | (2) |
| RLG14475 | MG1655 | (3) |
| RLG14718 | RLG6348 pTrc99a-empty vector, Amp^R^ | (4) |
| RLG14719 | RLG6348 pTrc99a-*traR*, Amp^R^ | (4) |
| RLG15137 | RLG6348 pTrc99a-*dksA,* Amp^R^ | This study |
| RLG15139 | RLG6348 pTrc99a- λ o*rf73,* Amp^R^ | This study |
| RLG15140 | RLG6348 pTrc99a- P2 o*rf82,* Amp^R^ | This study |
| RLG15141 | RLG6348 pTrc99a-*X.b gp34,* Amp^R^ | This study |
| RLG15162 | RLG6348 pTrc99a-VP882 o*rf61,* Amp^R^ | This study |
| RLG15169 | RLG6348 pTrc99a-VHML o*rf8,* Amp^R^ | This study |
| RLG15636 | MG1655 pBAD-empty vector, Amp^R^ | Gourse lab collection |
| RLG15592 | MG1655 pBAD-*traR*, Amp^R^ | This study |
| RLG15593 | MG1655 pBAD- λ *orf73*, Amp^R^ | This study |
| HME6 | W3110 Δ(*argF-lac*)*U*169 [ƛ*c*I857Δ(*cro-bioA*)] *galK_TYR145UAG_* | (5) |
| XTL241 | HME6 *cat*-*sacB*<>lambda *gam* | This study |
| RLG15323 | W3110 Δ(*argF-lac*)*U*169 [ƛ*c*I857Δ(*cro-bioA*) *CIII-orf73* fusion] *galK_TYR145UAG_* | This study |
| RLG15324 | W3110 Δ(*argF-lac*)*U*169 [ƛ*c*I857Δ(*cro-bioA*) *CIII-ea22* fusion] *galK_TYR145UAG_* | This study |
| RLG15333 | MG1655 pOX38-*traR*+MiniF, Kan^R^ | (6) |
| RLG15334 | MG1655 pOX38-Δ*traR* MiniF, Kan^R^ | (6) |
| XTL850 | MG1655 ƛ lysogen, single copy | This study |
| XTL1054 | XTL850 pKM208 | This study |
| XTL1055 | XTL1054 *orf73*<>*tet*-*sacB* | This study |
| RLG15500 | MG1655 λ *orf73* WT | This study |
| RLG15499 | MG1655 λ *orf73 (*ΔD3-S58) | This study |
| RLG15498 | MG1655 λ *orf73* D3A D6A | This study |
|  |  |  |
|  | | |
| **Plasmid** | **Description** | **Source** |
| pRLG770 | In vitro transcription vector, Amp^R^ | (7) |
| pRLG5073 | pRLG770 with *thrABC* (-72 to +16) | (8) |
| pRLG8150 | His_6_-HMK-DksA | (9) |
| pRLG13065 | pRLG770 with *rrnB* P1 (-88 to +50) | (7) |
| pRLG13098 | pRLG770 with *argI* (-45 to +32) | (8) |
| pRLG13068 | pRLG770 with *rpoHP3* | (10) |
| pRLG14658 | pRLG770 with *rpsT* P2 (-89 to +50) | (11) |
| pRLG14869 | pRLG770 with *dnaA* P1P2 (-165 to +135) | (3) |
| pRLG15510 | pRLG770 with λ p*L* (-150 to +50) | This study |
| pRLG15512 | pRLG770 with λ p*R* (-150 to +50) | This study |
| pRLG15509 | pRLG770 with λ p*RM* (-150 to +50) | This study |
| pRLG15513 | pRLG770 with λ p*R’* (-150 to +50) | This study |
| pRLG15298 | pRLG770 with p*Y* (-100 to +50) | This study |
| pRLG15297 | pRLG770 with p*traJ* (-100 to +50) | This study |
| pRLG15296 | pRLG770 with p*traM* (-100 to +50) | This study |
| pRLG15374 | pRLG770 with p*finP* (-100 to +50) | This study |
| pRLG15667 | pRLG770 with *V.cholerae* *rrnB* P1 (-150 to +50) | This study |
| pRLG15668 | pRLG770 with *V.cholerae* *rpsT* P2 (-100 to +50) | This study |
| pRLG15142 | pET28a-His_10_-Sumo-TraR, Kan^R^ | (12) |
| pRLG15381 | pET28a-His_10_-Sumo- λ Orf73, Kan^R^ | This study |
| pRLG15482 | pET28a-His_10_-Sumo-VP882 Orf61, Kan^R^ | This study |
| pRLG15097 | pET28a-VHML Orf8-His_6_, Kan^R^ | This study |
| pRLG15382 | pET28a-His_10_-Sumo-P2 Orf82, Kan^R^ | This study |
| pRLG15099 | pET28a-*X. bovenii* Gp34-His_6_, Kan^R^ | This study |
| pRLG15393 | pET28a-His_10_-Sumo- λ D3N-Orf73, Kan^R^ | This study |
| pRLG15394 | pET28a-His_10_-Sumo- λ D6N-Orf73, Kan^R^ | This study |
|  |  |  |

**Supplemental References**

1. Paul BJ, Barker MM, Ross W, Schneider DA, Webb C, Foster JW, Gourse RL. 2004. DksA: A critical component of the transcription initiation machinery that potentiates the regulation of rRNA promoters by ppGpp and the initiating NTP. Cell 118:311-322.

2. Blattner FR, Plunkett G 3rd, Bloch CA, Perna NT, Burland V, Riley M, Collado-Vides J, Glasner JD, Rode CK, Mayhew GF, Gregor J, Davis NW, Kirkpatrick HA, Goeden MA, Rose DJ, Mau B, Shao Y. 1997. The complete genome sequence of *Escherichia coli* K-12. Science 277(5331):1453-1462.

3. Sanchez-Vazquez P, Dewey CN, Kitten N, Ross W, Gourse RL (2019). Genome-wide effects of *Escherichia coli* transcription from ppGpp binding to its two sites on RNA polymerase Proc Natl Acad Sci USA 116(17):8310-8319.

4. Gopalkrishnan S, Ross W, Chen AY, Gourse RL. 2017. TraR directly regulates transcription initiation by mimicking the combined effects of the global regulators DksA and ppGpp. Proc Natl Acad Sci USA 114(28)e5539-5548.

5. [Ellis, HM, Yu D, DiTizio T, Court DL. 2001. High efficiency mutagenesis, repair, and engineering of chromosomal DNA using single-stranded oligonucleotides. Proc Natl Acad Sci USA 98(12):6742–6746.](http://paperpile.com/b/r5aM7n/0STr)

6. Blankschien MD, Potrykus K, Grace E, Choudhary A, Vinella D, Cashel M, Herman C. 2009. TraR, a homolog of a RNAP secondary channel interactor, modulates transcription. PLoS Genet 5(1):e1000345.

7. Ross W, Thompson JF, Newlands JT, Gourse RL. 1990. *E. coli* Fis protein activates ribosomal RNA transcription in vitro and in vivo. EMBO J 9:3733-3742.

8. Barker MM, Gaal T, Josaitis CA, Gourse RL. 2001. Mechanism of regulation of transcription initiation by ppGpp. I. Effects of ppGpp on transcription initiation in vivo and in vitro. J Mol Biol 305:673-688.

9. Lennon CW, Gaal T, Ross W, Gourse RL. 2009. *Escherichia coli* DksA binds to free RNA polymerase with higher affinity than to RNA polymerase in an open complex. J Bacteriol 191:5854-5858.

10. Costanzo A, Nicoloff H, Barchinger S, Banta A, Gourse RL, Ades SE. 2008. Mechanism of regulation of the extracytoplasmic stress factor σ^E^ in *Escherichia coli* by DksA and the alarmone ppGpp. Mol Microbiol 67:619-632.

11. Lemke JJ, Sanchez-Vazquez P, Burgos-Robles H, Hedberg G, Ross W, and Gourse RL. 2011. Direct regulation of *Escherichia coli* ribosomal protein promoters by ppGpp/DksA, Proc Natl Acad Sci USA 108:5712-5717.

12. Chen J, Gopalkrishnan S, Chiu C, Chen AY, Campbell EA, Gourse RL, Ross W, Darst SA. 2019. *E. coli* TraR allosterically regulates transcription initiation by altering RNA polymerase conformation. eLife 8:e49375.
