# Supplementary material for: Homologs of the Escherichia coli F Element Protein TraR, Including Phage Lambda Orf73, Directly Reprogram Host Transcription
Source: mBio. 2022 May 18;13(3):e00952-22. doi: 10.1128/mbio.00952-22 (PMC9239242; doi:10.1128/mbio.00952-22)
Supplement: TABLE S2 [file mbio.00952-22-s0006.docx]

**Table S2: Oligonucleotides**

F = forward primer, R = reverse primer

| **Primer Name or #** | **Sequence** | **Purpose** |
| --- | --- | --- |
| 7092 (F) | 5’ GAGCAAGCGGACCTCATAAA 3’ | RT-qPCR of 16S rRNA |
| 7093 (R) | 5’ GGCATTCTGATCCACGATTACTA 3’ |  |
| 7292 (F) | 5’ CTGAGTTGTTATTTAAGCTTGCC 3’ | RT-qPCR of *iraP* transcript |
| 7293 (R) | 5’ AAGCATTGCAGTGACGATAA 3’ |  |
| 7252 (F) | 5’ TGACACGGAACAACGGCAAACACG 3’ | RT-qPCR of rRNA leader |
| 7253 (R) | 5’ TGCATAATACGCCTTCCCGCTACA 3’ |  |
| 7057 (F) | 5′ AAGCTTGGGTCCCACCTGACC 3′ | Linearize transcription vector p770 |
| 7058 (R) | 5′ GAATTCTTGAAGACGAAAGGGCC 3′ |  |
| 4252 (R) | 5′ CCCGAAAAGTGCCACCTGACG 3′ | Sequence promoters cloned into p770 |
| 4253 (F) | 5′ CAGTTCCCTACTCTCGCATGG 3′ |  |
| 8666 (F) | 5’ ACCCACATCAGCACCAGTCAC 3’ | RT-qPCR of *katE* transcript |
| 8667 (R) | 5’ TCAGCCGCTGGACGATGAG 3’ |  |
| 8668 (F) | 5’ ACACATCAGATTTCCTGGTG 3’ | RT-qPCR of *dsrA* sRNA |
| 8669 (R) | 5’ GAAGTGAATCGTTGAATGCAC 3’ |  |
| 8672 (F) | 5’ AGAATTAAAGCTTGCTTCACTG 3’ | RT-qPCR of *dnaA* transcript |
| 8673 (R) | 5’ TGCCATTGAGCAGAGACTG 3’ |  |
| 8674 (F) | 5’ TATCAAATCAGCTAAGAAGCGC 3’ | RT-qPCR of *rpsT* transcript |
| 8675 (R) | 5’ TGCGTATACTTTCTTGATGAAAG 3’ |  |
| 8676 (F) | 5’ ACGCATTAGCACCACCATTAC 3’ | RT-qPCR of *thr* leader |
| 8677 (R) | 5’ AGGTGCGGGCTTTTTTCTGTG 3’ |  |
| 8680 (F) | CGGGTTTTATCATAAGCATTTCCTG | RT-qPCR of *argI* transcript |
| 8681 (R) | 5’ TTTGGCTTCTTCTTTACCGCT 3’ |  |
| Linear-FpBAD | 5’ AGCTTGGCTGTTTTGGCGGATGAGA 3’ | Linearize pBAD vector |
| Linear-RpBAD | 5’ GGTGAATTCCTCCTGTTAGCCCAAAA 3’ |  |
| pBAD_fwd_primer | 5’ ATGCCATAGCATTTTTATCC 3’ | Sequence promoters cloned into pBAD |
| pBAD_rev_primer | 5’ GATTTAATCTGTATCAGG 3’ |  |
| Orf73-D3N | 5’ CAGATTGGTGGAGCAAACATCATTGATTCAGCAT 3’ | Introduce site-directed mutation in pET28a-His_10_-Sumo- λ *orf73* |
| Orf73-D6N | 5’ TGGAGCAGACATCATTAATTCAGCATCAGAAATA 3’ |  |
| SumoForwardSeq | GGGGAATTGTGAGCGGATAACAATTCC | Sequence insert cloned into pET28a-His_10_-Sumo vector |
| SumoReverseSeq | GTCCCATTCGCCAATCCGGATATAG |  |
