# Supplementary material for: Homologs of the Escherichia coli F Element Protein TraR, Including Phage Lambda Orf73, Directly Reprogram Host Transcription
Source: mBio. 2022 May 18;13(3):e00952-22. doi: 10.1128/mbio.00952-22 (PMC9239242; doi:10.1128/mbio.00952-22)
Supplement: TABLE S3 [file mbio.00952-22-s0007.docx]

**Table S3** Oligonucleotides used in construction of λ phage variants

| Oligo# | Oligo sequence | Role |
| --- | --- | --- |
| XT409 | aagaggcagaactggcagacgacatggaaaaaggcctgccccagcacctgTGTGACGGAAGATCACTTCGCAG | *cat*/*sacB*<>*gamma* |
| XT410 | tttttgctggccccgtggcgttgcaaatgatcgatgcatagcgattcaaaATCAAAGGGAAAACTGTCCATAT | *cat*/*sacB*<>*gamma* |
| XT415 | cgaggcgtttttcgttatgtataaataaggagcacaccatggcagacatcattgattcagcatcagaaatagaaga | *CIII-orf73* fusion |
| XT419 | cgaggcgtttttcgttatgtataaataaggagcacaccgtgagcgaaattaactctcaggcactgcgtgaagc | *CIII-ea22* fusion |
| XT1028 | TTTATTGCTGTGTTGCGCTGTAATTCTTCTATTTCTGATGCTGAATCAATATCAAAGGGAAAACTGTCCATATGC | *Orf73*<>*Tet-sacB* |
| XT1029 | TCAAGCGTATTAACCAACAGTTCAGGGATTAATGAAAGATGGCAGACATCTCCTAATTTTTGTTGACACTCTATC | *orf73*<>*Tet-sacB* |
| XT1032 | TAACCAACAGTTCAGGGATTAATGAAAGATGGCAGCCATCATTGCTTCAGCATCAGAAATAGAAGAATTACAGCGCAACA | *orf73* D3A D6A |
| XT1033 | TTAACCAACAGTTCAGGGATTAATGAAAGATGGCAAGTTGCCAGGAGGATCTGGAACTTATCAGTAAACA | *orf73* Δ(D3-S58) |
| XT1043 | TAACCAACAGTTCAGGGATTAATGAAAGATGGCAGACATCATTGATTCAGCATCAGAAATAGAAGAATTACAGCGCAACA | *orf73* WT |
