# Supplementary material for: Homologs of the Escherichia coli F Element Protein TraR, Including Phage Lambda Orf73, Directly Reprogram Host Transcription
Source: mBio. 2022 May 18;13(3):e00952-22. doi: 10.1128/mbio.00952-22 (PMC9239242; doi:10.1128/mbio.00952-22)
Supplement: TABLE S4 [file mbio.00952-22-s0008.docx]

**Table S4.** Geneblocks (start and stop codons, promoter elements, and transcription start sites in bold and underlined)

| **gBlock** | **Sequence** |
| --- | --- |
| pBAD-*traR* | GGATCCTACCTGACGCTTTTTATCGCAACTCTCTACTGTTTCTCCATACCCGTTTTTTGGGCTAACAGGAGGAATTCACC**ATG**AGTGATGAAGCCGATGAAGCATATTCAGTGACAGAACAACTGACCATGACAGGAATAAACCGGATACGCCAGAAAATAAATGCTCATGGTATTCCTGTTTATCTCTGTGAAGCATGCGGAAATCCTATTCCGGAAGCCCGGCGGAAAATATTTCCCGGTGTGACGTTGTGCGTTGAATGTCAGGCGTATCAGGAAAGACAGAGAAAACATTATGCA**TAA**AGCTTGGCTGTTTTGGCGGATGAGAGAAGATTTTCAGCCTGATACAGATTAAATCAGAACGCAGAAGCGGTCTGATAAAA |
| pBAD-*orf73* | GGATCCTACCTGACGCTTTTTATCGCAACTCTCTACTGTTTCTCCATACCCGTTTTTTGGGCTAACAGGAGGAATTCACC**ATG**GCAGACATCATTGATTCAGCATCAGAAATAGAAGAATTACAGCGCAACACAGCAATAAAAATGCGCCGCCTGAACCACCAGGCTATATCTGCCACTCATTGTTGTGAGTGTGGCGATCCGATAGATGAACGAAGACGCCTGGTCGTTCAGGGTTGTCGGACTTGTGCAAGTTGCCAGGAGGATCTGGAACTTATCAGTAAACAGAGAGGTTCGAAG**TAA**AGCTTGGCTGTTTTGGCGGATGAGAGAAGATTTTCAGCCTGATACAGATTAAATCAGAACGCAGAAGCGGTCTGATAAAA |
| pTrc-*dksA* | ataacaAtttcacacaggaaacagaccatggaattcCGTGTCAGAACATGTCGGGAGGAATATTCC**ATG**CAAGAAGGGCAAAACCGTAAAACATCGTCCCTGAGTATTCTCGCCATCGCTGGGGTGGAACCATATCAGGAGAAGCCGGGCGAAGAGTATATGAATGAAGCCCAGCTGGCGCACTTCCGTCGTATTCTGGAAGCATGGCGTAATCAACTCAGGGATGAAGTCGATCGCACCGTTACACATATGCAGGATGAAGCAGCCAACTTCCCGGACCCGGTAGACCGTGCAGCCCAGGAAGAAGAGTTCAGCCTCGAACTGCGTAACCGCGATCGCGAGCGTAAGCTGATCAAAAAGATCGAGAAGACGCTGAAAAAAGTGGAAGACGAAGATTTCGGCTACTGCGAATCCTGCGGTGTTGAAATTGGTATTCGCCGTCTGGAAGCGCGCCCGACAGCCGATCTGTGCATCGACTGCAAAACGCTGGCTGAAATTCGCGAAAAACAGATGGCTGGC**TAA**GTCAGTCGCAGAACATAGTGAAAGCTTggctgttttggcggatgagagaagattttc |
| pTrc- λ *orf73* | ataacaAtttcacacaggaaacagaccatggaattcCGTGTCAGAACATGTCGGGAGGAATATTCC**ATG**GCAGACATCATTGATTCAGCATCAGAAATAGAAGAATTACAGCGCAACACAGCAATAAAAATGCGCCGCCTGAACCACCAGGCTATATCTGCCACTCATTGTTGTGAGTGTGGCGATCCGATAGATGAACGAAGACGCCTGGTCGTTCAGGGTTGTCGGACTTGTGCAAGTTGCCAGGAGGATCTGGAACTTATCAGTAAACAGAGAGGTTCGAAG**TGA**GTCAGTCGCAGAACATAGTGAAAGCTTggctgttttggcggatgagagaagattttc |
| pTrc-P2 *orf82* | ataacaAtttcacacaggaaacagaccatggaattcCGTGTCAGAACATGTCGGGAGGAATATTCC**atg**ccggacaacgtagattttattcaggaacaacaggctgaattactggagcgccagattaacgcggcaagggtaaaacattgcggtgtttctgcgctggtttgcgaagagtgtgacgcgccaatacctgctgtccgtcgtgcagcttatccgtcagccacgcgttgtgtttcctgccagtcagtctttgaagcaaaaaacaagcattaccggagaatggca**TGA**GTCAGTCGCAGAACATAGTGAAAGCTTggctgttttggcggatgagagaagattttc |
| pTrc-*X.b gp34* | ataacaAtttcacacaggaaacagaccatggaattcCGTGTCAGAACATGTCGGGAGGAATATTCC**atg**tctaaagcgctcgacctcgccattcagcacgttgatgaaatgctggaacgccggatagccgcacatgttaaccgccctgtcggcgtttctgcttttaagtgtgagagctgcggaaatcccatccctgagcaacgtcgaatgattattgcaggcgtgaccctttgtgcgccttgccagaacgtttttgaactgaaacagaaacattaccggagtgag**TGA**GTCAGTCGCAGAACATAGTGAAAGCTTggctgttttggcggatgagagaagattttc |
| pTrc-VHML *orf8* | ATAACAATTTCACACAGGAAACAGACCATGGAATTCCGTGTCAGAACATGTCGGGAGGAATATTCC**ATG**ACTGACTGCAGTGCTGACCCGTTAGACAGAGCCGCTGCTCTTTCTCAAGCACACCTTGAGGTGTCCTTGTCTCGGATTAAGAAGTTTGAGGGCGTATCAGCCCATGAGTGTGTTGAATGTGGGTCTGAAATACCGAAGAAGCGGCGAGAGCTTTTGCAGGGTGTAACCGACTGTGTTGACTGTGCAGCAATCAAAGAAACATTAAGCAAGAACTACATGCGA**TAG**GTCAGTCGCAGAACATAGTGAAAGCTTGGCTGTTTTGGCGGATGAGAGAAGATTTTC |
| pTrc-VP882 *orf61* | ATAACAATTTCACACAGGAAACAGACCATGGAATTCCGTGTCAGAACATGTCGGGAGGAATATTCC**ATG**AGTGATATTGCAGATCAGGCACAGGACGTCATCGAGCAGCACCTGACGGCCAGCCTGGCGAACAGGAAGCACAACATCAACCCAGCCATCCCAAGCGCGAAGCATTGCGATGACTGCGAGTCGGAAATCCCAGAGGCTCGCCGTCGCAGTCTTCCTGGTGTCCGCTTGTGTGTTGATTGCGCTTCTCTGCAGGAGATTAAAGGCCGACATCAAAGG**TAG**GTCAGTCGCAGAACATAGTGAAAGCTTGGCTGTTTTGGCGGATGAGAGAAGATTTTC |
| p770-*Vc*-*rrnA/B* P1 | GGCCCTTTCGTCTTCAAGAATTCcccttttataagcaagtggtaggtatagtagatggcttaatcattaacaaatagcctgttctgcctaaaacatcaccaagtgctcgaaaactttaaaaaacttctaaaaagtac**ttgcca**atataactaggttctc**tataat**ccgccctc**a**ctgatacggcagacgcagtaagcgttagcagtgattcagctcggtgtttAAGCTTGGGTCCCACCTGACCC |
| p770-*Vc*-*rpsT* P2 | GGCCCTTTCGTCTTCAAGAATTCtcttaaccgcgaaggctcttcgataccaagtttgtttggtccagttgggtttttgcacaaatcat**ttgaca**ttaaagagcaaatcggg**gatatt**tcccgc**c**cttaaaatgtcaccgaactaagtttttgggagttagacccttggcaaatAAGCTTGGGTCCCACCTGACCC |
| p770-p*traM* | GGCCCTTTCGTCTTCAAGAATTCCACCGTTTTGTAGGGGTGGTACTGACTATTTTTATAAAAAACATTATTTTATATTAGGGGTG**CTGCTA**GCGGCGCGGTGTGTTTTTTTA**TAGGAT**ACCGC**T**AGGGGCGCTGCTAGCGGTGCGTCCCTGTTTGCATTATGAATTTTAGTGTAAGCTTGGGTCCCACCTGACCC |
| p770-p*traJ* | GGCCCTTTCGTCTTCAAGAATTCAAAAAATGATGATGAATAAACGAAATTTGACTTCGTTCAAATATCAGAGTTTTTATGATTTAAAAAG**GTGACA**GTACGAAAGATAAT**TAGTAT**ATTAATT**A**CGTGGTTAATGCCACGTTAAAATTTGAAATTGAAAATCGCCGATGCAGGAAGCTTGGGTCCCACCTGACCC |
| p770-p*Y* | GGCCCTTTCGTCTTCAAGAATTCGAAGGCTATGTGTATCATAAATACGCGTTAATAAGGTGTTAATAAAATATAGACTTTCCGTCTA**TTTACC**TTTTCTGATTATTCTGC**AAACAT**AAGTGGT**A**ACCAGAAGATAAACAGCGGGAGGTGTTATTGAAAAGATTTGGTACACGTAAGCTTGGGTCCCACCTGACCC |
| p770-p*finP* | GGCCCTTTCGTCTTCAAGAATTCTCCCTGATACAGGCTGGATTTGGATAATCCTGAATAACTGCCGTCAGATTTTCCAGCAGATCTAT**TTGACG**AGCATGTTTTTGTTGAA**TACGAT**CCATCG**G**ATACATAGGAACCTCCTCACAAAGGATTCTATGGACAGTCGATGCAGGGAAGCTTGGGTCCCACCTGACCC |
| p770-pL | GGCCCTTTCGTCTTCAAGAATTCCTAAACATAGCAATTCAGATCTCTCACCTACCAAACAATGCCCCCCTGCAAAAAATAAATTCATATAAAAAACATACAGATAACCATCTGCGGTGATAAATTATCTCTGGCGGTG**TTGACA**TAAATACCACTGGCGGT**GATACT**GAGCAC**A**TCAGCAGGACGCACTGACCACCATGAAGGTGACGCTCTTAAAAATTAAGAAGCTTGGGTCCCACCTGACCC |
| p770-pR | GGCCCTTTCGTCTTCAAGAATTCTAAATTGCTTTAAGGCGACGTGCGTCCTCAAGCTGCTCTTGTGTTAATGGTTTCTTTTTTGTGCTCATACGTTAAATCTATCACCGCAAGGGATAAATATCTAACACCGTGCGTG**TTGACT**ATTTTACCTCTGGCGGT**GATAAT**GGTTGC**A**TGTACTAAGGAGGTTGTATGGAACAACGCATAACCCTGAAAGATTATGCAAGCTTGGGTCCCACCTGACCC |
| p770-pRM | GGCCCTTTCGTCTTCAAGAATTCGTTTGCCCAAAGCGCATTGCATAATCTTTCAGGGTTATGCGTTGTTCCATACAACCTCCTTAGTACATGCAACCATTATCACCGCCAGAGGTAAAATAGTCAACACGCACGGTGT**TAGATA**TTTATCCCTTGCGGTGA**TAGATT**TAACGT**A**TGAGCACAAAAAAGAAACCATTAACACAAGAGCAGCTTGAGGACGCACGAAGCTTGGGTCCCACCTGACCC |
| p770-pR’ | GGCCCTTTCGTCTTCAAGAATTCTGACGCTCTGGTGGTGCAATGCCACAAAGAAGAGTCAATCGCAGACAACATTTTGAATGCGGTCACACGTTAGCAGCATGATTGCCACGGATGGCAACATATTAACGGCATGATA**TTGACT**TATTGAATAAAATTGGG**TAAATT**TGACTC**A**ACGATGGGTTAATTCGCTCGTTGTGGTAGTGAGATGAAAAGAGGCGGCGAAGCTTGGGTCCCACCTGACCC |
| pET28a-His_10_-Sumo-λ *orf73* | CTGAAGATTTGGACATGGAGGATAACGATATTATTGAGGCTCACAGAGAACAGATT**GGTGGA**GCAGACATCATTGATTCAGCATCAGAAATAGAAGAATTACAGCGCAACACAGCAATAAAAATGCGCCGCCTGAACCACCAGGCTATATCTGCCACTCATTGTTGTGAGTGTGGCGATCCGATAGATGAACGAAGACGCCTGGTCGTTCAGGGTTGTCGGACTTGTGCAAGTTGCCAGGAGGATCTGGAACTTATCAGTAAACAGAGAGGTTCGAAG**TGA**CAAAGCCCGAAAGGAAGCTGAGTTGGCTGCTGCCACCGCTGAGCAATAACTAGCATAACCCC |
| pET28a-His_10_-Sumo-P2 *orf82* | CTGAAGATTTGGACATGGAGGATAACGATATTATTGAGGCTCACAGAGAACAGATT**GGTGGA**ccggacaacgtagattttattcaggaacaacaggctgaattactggagcgccagattaacgcggcaagggtaaaacattgcggtgtttctgcgctggtttgcgaagagtgtgacgcgccaatacctgctgtccgtcgtgcagcttatccgtcagccacgcgttgtgtttcctgccagtcagtctttgaagcaaaaaacaagcattaccggagaatggca**TGA**CAAAGCCCGAAAGGAAGCTGAGTTGGCTGCTGCCACCGCTGAGCAATAACTAGCATAACCCC |
| pET28a-His_10_-Sumo-VP882 *orf61* | CTGAAGATTTGGACATGGAGGATAACGATATTATTGAGGCTCACAGAGAACAGATT**GGTGGA**agtgatattgcagatcaggcacaggacgtcatcgagcagcacctgacggccagcctggcgaacaggaagcacaacatcaacccagccatcccaagcgcgaagcattgcgatgactgcgagtcggaaatcccagaggctcgccgtcgcagtcttcctggtgtccgcttgtgtgttgattgcgcttctctgcaggagattaaaggccgacatcaaagg**TAG**CAAAGCCCGAAAGGAAGCTGAGTTGGCTGCTGCCACCGCTGAGCAATAACTAGCATAACCCC |
| pET28a- *X.b Gp34-His_6_* | GTGAGCGGATAACAATTCCCCTCTAGAAATAATTTTGTTTAACTTTAAGAAGGAGATATACC**atg**tctaaagcgctcgacctcgccattcagcacgttgatgaaatgctggaacgccggatagccgcacatgttaaccgccctgtcggcgtttctgcttttaagtgtgagagctgcggaaatcccatccctgagcaacgtcgaatgattattgcaggcgtgaccctttgtgcgccttgccagaacgtttttgaactgaaacagaaacattaccggagtgagCTGGTGCCGCGTGGCAGCAGCAGCGGCCACCATCACCATCACCAT**TGA**TGAGATCCGGCTGCTAACAAAGC |
| pET28a-VHML-Orf8-His_6_ | GTGAGCGGATAACAATTCCCCTCTAGAAATAATTTTGTTTAACTTTAAGAAGGAGATATACCatgactgactgcagtgctgacccgttagacagagccgctgctctttctcaagcacaccttgaggtgtccttgtctcggattaagaagtttgagggcgtatcagcccatgagtgtgttgaatgtgggtctgaaataccgaagaagcggcgagagcttttgcagggtgtaaccgactgtgttgactgtgcagcaatcaaagaaacattaagcaagaactacatgcgaCTGGTGCCGCGTGGCAGCAGCAGCGGCCACCATCACCATCACCAT**TAG**TGAGATCCGGCTGCTAACAAAGC |
